# PERM1 interacts with the MICOS-MIB complex to connect the mitochondria and sarcolemma via ankyrin B

**DOI:** 10.1038/s41467-021-25185-3

**Published:** 2021-08-12

**Authors:** Theresa Bock, Clara Türk, Sriram Aravamudhan, Lena Keufgens, Wilhelm Bloch, Dieu Hien Rozsivalova, Vanina Romanello, Leonardo Nogara, Bert Blaauw, Aleksandra Trifunovic, Thomas Braun, Marcus Krüger

**Affiliations:** 1 Institute for Genetics, Cologne Excellence Cluster on Cellular Stress Responses in Aging-Associated Diseases (CECAD), Cologne, Germany; 2 BASF SE, Metabolomics and Proteomics, Ludwigshafen am Rhein, Germany; 3 Cell Signaling Technology, Leiden, Netherlands; 4 Department of Molecular and Cellular Sport Medicine, Institute of Sport Medicine and Cardiovascular Research, German Sport University Cologne, Cologne, Germany; 5 Venetian Institute of Molecular Medicine (VIMM), Department of Biomedical Sciences Padova, University of Padova, Padova, Italy; 6 Center for Molecular Medicine (CMMC), University of Cologne, Cologne, Germany; 7 Max Planck Institute for Heart and Lung Research, Bad Nauheim, Germany

**Keywords:** Cytoskeleton, Mitochondria, Proteomics, Protein-protein interaction networks

## Abstract

Skeletal muscle subsarcolemmal mitochondria (SSM) and intermyofibrillar mitochondria subpopulations have distinct metabolic activity and sensitivity, though the mechanisms that localize SSM to peripheral areas of muscle fibers are poorly understood. A protein interaction study and complexome profiling identifies PERM1 interacts with the MICOS-MIB complex. Ablation of *Perm1* in mice reduces muscle force, decreases mitochondrial membrane potential and complex I activity, and reduces the numbers of SSM in skeletal muscle. We demonstrate PERM1 interacts with the intracellular adaptor protein ankyrin B (ANKB) that connects the cytoskeleton to the plasma membrane. Moreover, we identify a C-terminal transmembrane helix that anchors PERM1 into the outer mitochondrial membrane. We conclude PERM1 functions in the MICOS-MIB complex and acts as an adapter to connect the mitochondria with the sarcolemma via ANKB.

## Abstract (web summary)

Mitochondria in skeletal muscle have distinct localization and properties through unclear mechanisms. Here, the authors use complexome profiling and immunoprecipitations to identify PERM1 as a MICOS-MIB complex interactor that also binds ankyrin B, suggesting PERM1 directs the mitochondria to the membrane.

## Introduction

Skeletal muscle possesses remarkable plasticity and can rapidly adapt to environmental factors such as workload, nutrient supply and neuronal stimulation^1^. For example, physical activity increases the number of mitochondria and their oxidative capacity. Moreover, muscle activity improves the regeneration of damaged muscles and promotes vascularization of the skeletal muscles^2^. Previous studies revealed two mitochondrial subpopulations: subsarcolemmal mitochondria (SSM), located below the sarcolemmal membrane, and interfibrillar mitochondria (IFM), situated between myofibrils^3^. IFM are smaller and have a more compact shape, while SSM are larger and own a lamellar structure. High-resolution electron microscopy revealed the existence of additional mitochondrial subpopulations^4^, which may also differ in their protein composition, respiration, and sensitivity to metabolic changes^5^. Studies of cardiac mitochondria demonstrated that SSM are more susceptible to cellular stress, such as ischemic injury and calcium overload, than IFM^6^. However, the mechanisms that determine the position and regulate the activity of different mitochondrial subpopulations remain largely unknown.

Activation of mitochondrial gene expression depends on the key transcriptional coactivators peroxisome proliferator-activated receptor γ coactivator 1a (PGC-1α) and the estrogen-related receptor α (ERRα)^7^. Enhanced muscle workload upregulates PGC-1α activity and promotes mitochondrial biogenesis^8^. Conversely, loss of function of PGC-1α results in defects in cardiac and skeletal muscle energetics and causes cardiomyopathy^9^. Similarly, muscle workload increases the intracellular calcium ion concentration and activates the Ca^2+^/calmodulin-dependent protein kinase II (CaMKII) and protein kinase p38. Activation of this cascade increases PGC-1α protein expression and mitochondrial biogenesis^10^. Additionally, PGC-1α-dependent transcription factors, such as CREB and MEF2, are activated via a feed-forward loop^11^. PGC-1- and ERR-induced regulator in muscle 1 (PERM1) was recently identified to regulate the expression of PGC-1α and ERRα targets to enhance mitochondrial biogenesis and adaptive oxidative metabolism in skeletal muscle^12^. PERM1 protein expression is mainly restricted to heart and skeletal muscle, indicating PERM1 functions as a tissue-specific regulator of mitochondrial biogenesis in response to exercise. *Perm1* loss- and gain-of-function experiments in cell culture^12^ and adenoviral-mediated overexpression in mouse muscles revealed that PERM1 regulates muscle-related pathways modulating muscle contraction, vascularization and metabolic activity^13^.

PERM1 has been detected in the nucleus and the cytosol^12^ but also in the outer mitochondrial membrane (OMM)^14^. Casein kinase (CK2)-mediated phosphorylation of the PEST motif leads to degradation of PERM1^14^. However, little is known about the mechanisms by which PERM1 increases oxidative capacity and mitochondrial biogenesis. A previous study showed that PERM1 interacts with CaMKII and induced phosphorylation of p38^15^. In addition, PERM1 regulates *Gadd45g* and *Nr4A3*, possibly also through CaMKII, which are involved in the control of exercise-responsive gene expression and oxidative capacity.

The mitochondrial contact site and cristae organizing system (MICOS) complex is involved in the regulation of mitochondrial metabolic activity^16^ and plays an important role for the organization of the inner mitochondrial membrane (IMM) and cristae formation. It is composed of MIC10 (MINOS1), MIC13 (QIL1), MIC19 (CHCHD3), MIC25 (CHCHD6), MIC26 (APOO), MIC27 (APOOL) and MIC60 (Mitofilin; IMMT)^17–20^. The MICOS core complex localizes to the inner mitochondrial membrane and closely interacts with the proteins that form the mitochondrial intermembrane space bridging (MIB) complex, which connects the inner and outer mitochondrial membrane. The MIB complex partially localizes in the OMM, thereby constituting the sorting and assembly machinery (SAM), which associates with other OMM proteins, such as channel proteins, components of the protein translocases, and mitochondrial fusion machines. Notably, MIC60, a central subunit of the MICOS complex, interacts with all known MICOS and MIB members. Depletion of MIC60 leads to a severe loss of cristae structures and reduced oxidative capacity^21^. Moreover, the stability of the MICOS–MIB complex is directly connected to biogenesis and assembly of respiratory chain complexes. For example, SAM50 is a critical stabilizing protein of the MIB complex responsible for protein transport, assembly of respiratory chain complexes and regulation of cristae integrity^22^.

In this study, we demonstrate that genetic ablation of *Perm1* in mice reduces the number of mitochondria in peripheral areas of muscle fibers, which compromises running capacity and skeletal muscle force production. Comprehensive mass spectrometry-based mapping of protein-protein interactions revealed that PERM1 interacts with several members of the MICOS–MIB complex and cytoskeletal adaptor proteins such as vimentin and ankyrin B. Native gel electrophoresis in combination with mass spectrometry demonstrated PERM1 co-migrated with the mature MICOS–MIB complex at 2.2 MDa. Moreover, deletion of a predicted transmembrane helix in the C-terminus of PERM1 led to specific loss of the interaction with MICOS-MIB proteins.

## Results

### Deletion of *Perm1* reduces muscle force without morphometric changes in skeletal muscle architecture

**Fig. 1:**
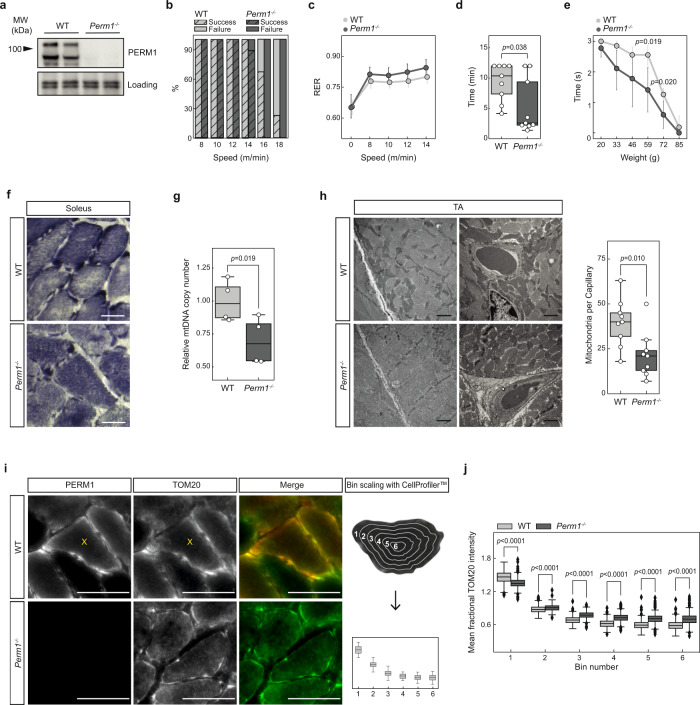
Deletion of *Perm1* reduces muscle force and alters the localization of subsarcolemmal mitochondria. **a** Western blot depicting successful depletion of *Perm1* in *Perm1*^−/−^ TA muscle. Correct genotype was confirmed by western blotting for each mouse used in the whole study (*n* > 20 mice per group). **b**, **c** Forced running experiments with metabolic respiratory exchange ratio (RER) measurements. **b** Percentage of wild-type and *Perm1*^*−/−*^ mice that succeeded or failed to run at the indicated speed for two minutes each (*n* = 9 mice per group). **c** RER (VCO_2_/VO_2_) of wild-type and *Perm1*^*−/−*^ mice running at the indicated speed (*n* = 9 mice per group). **d** Inverted screen test; the time *Perm1*^*−/−*^ and wild-type mice held their body weight on an inverted grid is shown (*n* = 9 mice per group). **e** Weight test; the time *Perm1*^*−/−*^ and wild-type mice grasped increasing weights is shown (*n* = 9 mice per group). **f** Succinate dehydrogenase (SDH) of soleus muscle from *Perm1*^*−/−*^ mice indicated an altered overall distribution of mitochondria compared to wild-type control. Scale 25 µm. **g** qPCR analysis showed decreased in mtDNA copy number in *Perm1*^*−/−*^ soleus muscle compared to wild-type controls (*n* = 3 mice per group). **h** Left: Electron microscopic inspection of TA muscle showed decreased mitochondrial density at capillaries in *Perm1*^*−/−*^ mice compared to wild-type mice. Scale 1 µm. Right: Number of mitochondria per capillary in *Perm1*^*−/−*^ and wild-type mice (*n* = 2 mice per group, 10 quantified capillaries). **i** Immunohistochemical staining of soleus muscle indicated co-localization of PERM1 (red) with TOM20 (green). Scale 50 µm. Schematic visualization of bin scaling of a single muscle fiber for TOM20 intensity quantification. **j** Mean fractional intensity of TOM20 signals in bins 1–6 from *Perm1*^*−/−*^ and wild-type soleus muscle fibers. Intensity is reduced at subsarcolemmal sites and increased within interfibrillar mitochondria in *Perm1*^*−/−*^ muscles compared to wild-type control (*n* = 2 mice per group, 507 quantified fibers). Data (**c**, **e**) are presented as mean values ±0.95 CI. Box plots (**d**, **g**, **h**, **j**) represent the median, 25th, and 75th percentiles, maximum and minimum are connected through whiskers, and individual data points are added on top (**d**, **g**, **h**). Outliers are defined as Q_1_− 1.8 interquartile range (IQR) and Q_3_+1.8 IQR. (**c**–**e**, **g**, **h**) unpaired two-sided Student’s *t* test, (**j**) unpaired two-sided Mann–Whitney *U*-test. Source data are provided as Source Data file.

To gain further insight into the physiological role of PERM1, we ablated *Perm1* in the mouse by replacing the coding region of exon 2 with a GFP cassette as described in^14^. Successful deletion of *Perm1* was confirmed by immunoblotting (Fig. 1a). *Perm1*^*−/−*^ mice are viable and fertile without obvious changes in body and muscle weight. Hematoxylin and eosin staining showed no obvious changes in *Perm1*^−/−^ muscle fibers (SI Fig. 1a). Immunostaining using antibodies directed against PERM1 and the heavy myosin chains (*Myh7, Myh2*) demonstrated that PERM1 is enriched within the subsarcolemmal regions in type IIa fibers, which contain higher numbers of mitochondria compared to slow type I and fast type IIb fibers. (SI Fig. 1b). Notably, the abundance of PERM1 correlated with the abundance of PCG-1α, which is highest in type IIa muscle fibers^23^. Detailed morphometric analysis of various fiber types in tibialis anterior (TA), extensor digitorum longus (EDL), and soleus muscle revealed no obvious fiber-type switches or changes in fiber diameter (SI Fig. 1c). Immunohistochemical staining using a CD31/PECAM1 antibody did not reveal any obvious alterations in the vascularization of *Perm1*^*−/−*^ skeletal muscles (SI Fig. 1d).

Next, we subjected *Perm1*^*−/−*^ and wild-type control littermates to forced running experiments and tested muscle strength. Treadmill exercise with increasing speed revealed no differences between mutants and wild-type controls up to moderate velocity of 12 m/min (Fig. 1b). However, *Perm1*^−/−^ mutants did not withstand 16 m/min for two minutes, while 70% of control mice endured this stress for two minutes. The respiratory exchange ratio (RER) was not altered between *Perm1*^*−/−*^ and control animals, indicating no significant changes in fuel sources (Fig. 1c). Assessment of limb muscle performance using an inverted screen test revealed a significantly shorter hold time for *Perm1*^*−/−*^ mutants compared to wild-type controls (Fig. 1d). In another muscle strength test, mice were lifted by the tail and given a fine-meshed net supplemented with a range of weights. *Perm1*^*−/−*^ mice had significantly reduced ability to carry weights from 59 g upwards compared to controls (Fig. 1e). Next, we measured the force generated by the gastrocnemius muscle in living animals after electrical stimulation. *Perm1*^*−/−*^ exhibited reduced strength compared to controls at all stimulation frequencies, although the differences did not reach statistical significance (*p*-value 0.09; SI Fig. S1e). Overall, the running performance, muscle strength and in vivo muscle stimulation experiments demonstrated that loss of PERM1 leads to reduced muscle strength and endurance. Thus, it will be interesting to investigate effects of *Perm1* deletion on force development in individual muscle groups with different fiber types and explore how PERM1 affects mitochondrial activity under normal and exercise conditions.

### Loss of *Perm1* reduces formation of SSM

Next, we visualized the enzymatic activity of the mitochondrial succinate dehydrogenase (SDH) in soleus muscle cross-sections. SDH signals were mainly detected in subsarcolemmal areas, reflecting the higher density of mitochondria at the periphery where the diffusion distances for oxygen and metabolites are shorter^24^. Importantly, we observed decreased SDH signal intensities at subsarcolemmal areas in *Perm1*^*−/−*^ fibers and a shift towards increased intensity in central areas (Fig. 1f). mtDNA copy number was lower in *Perm1*^*−/−*^ compared to wild-type control soleus muscle, suggesting a reduced mitochondrial content (Fig. 1g). Electron microscopy (EM) analysis of mitochondrial morphology revealed no changes in mitochondrial size and cristae formation (Fig. 1h, SI Fig. S2a). Consistent with the SDH staining, we observed the reduced formation of SSM in *Perm1*^*−/−*^ mutants, particularly close to capillaries (Fig. 1h).

To quantify mitochondrial distribution more precisely, we stained for TOM20, which co-localized with PERM1 at the periphery of muscle fibers, and determined the staining intensity from the periphery to the center of the muscle fibers. (Fig. 1i). We divided the cross-sectional area of single muscle fibers into six scaled bins and quantified the mean fractional TOM20 intensity for more than 500 fibers (*n* = 2). Since we observed a significant reduction in TOM20 in the first bin, next to the sarcolemma, in *Perm1*^*−/−*^ mutants compared to wild-type control sections (Fig. 1j), we hypothesize that inactivation of *Perm1* specifically reduces SSM, without affecting the IFM population.

### Loss of *Perm1* reduces the abundance of the MICOS/MIB complex in SSM

**Fig. 2:**
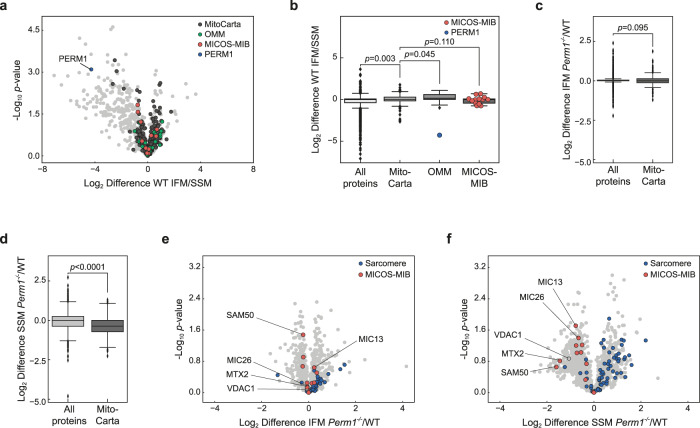
PERM1 is specifically enriched in SSM, and SSM are altered in *Perm1*^*−/−*^ muscles. **a**, **b** Volcano plot (**a**) and boxplot (**b**) depicting the regulation of PERM1, MitoCarta-annotated, OMM, and MICOS–MIB proteins in isolated IFM and SSM from wild-type TA muscle. Mitochondrial and OMM proteins are enriched in IFM. Inversely, PERM1 is clearly more abundant in the SSM fraction (*n* = 3 mice per group). **c**, **d** Comparison of log_2_ differences in MitoCarta 2.0 proteins compared to the complete dataset revealed no changes in the overall regulation of mitochondrial proteins in (**c**) isolated IFM of *Perm1*^*−/−*^ TA muscle compared to wild-type control, though MitoCarta-annotated proteins were significantly reduced in (**d**) isolated SSM from *Perm1*^*−/−*^ TA muscle compared to wild-type control (*n* = 3 mice per group). **e**, **f**) Volcano plots highlighting the regulation of sarcomeric proteins and proteins of the MICOS–MIB complex in (**e**) isolated IFM from *Perm1*^*−/−*^ TA compared to wild-type control and in (**f**) isolated SSM from *Perm1*^*−/−*^ TA compared to wild-type control (*n* = 3 mice per group). Box plots (**b**, **c**, **d**, **e**) represent the median, 25th, and 75th percentiles, maximum and minimum are connected through whiskers. Outliers are defined as Q_1_–1.8 IQR and Q_3_+1.8 IQR. **b**–**d** unpaired two-sided Mann-Whitney *U*-test. Source data are provided in Supplementary Data 1.

Since *Perm1* ablation affected the localization of SSM, we investigated whether PERM1 protein expression is more abundant in SSM than IFM in wild-type TA muscles. Consistent with the immunohistochemistry, we observed a higher PERM1 protein expression in SSM than IFM, while the majority of mitochondrial proteins, including OMM and MICOS–MIB members, were evenly distributed (Supplementary Data 1; Fig. 2a, b). We next compared SSM and IFM populations from *Perm1*^*−/−*^ and wild-type control mice. While we observed almost no protein changes in the IFM population (Fig. 2c), analysis of SSM mitochondria identified 62 regulated proteins, including several OMM and MICOS–MIB complex members (Fig. 2d–f). It should be noted that more sarcomeric proteins were detected in the *Perm1*^−/−^ SSM population compared to wild-type SSM, indicating altered associations of *Perm1*-deficient SSM with cytoskeletal and sarcomeric structures (Fig. 2f, blue circles).

Overall, the quantitative proteomics demonstrated that SSM are significantly more affected by *Perm1* ablation than IFM and that loss of PERM1 affects the protein expression of members of the MICOS–MIB complex.

### Inactivation of *Perm1* affects mitochondrial proteins related to transportation and breakdown of reactive oxygen species in skeletal muscle tissue

**Fig. 3:**
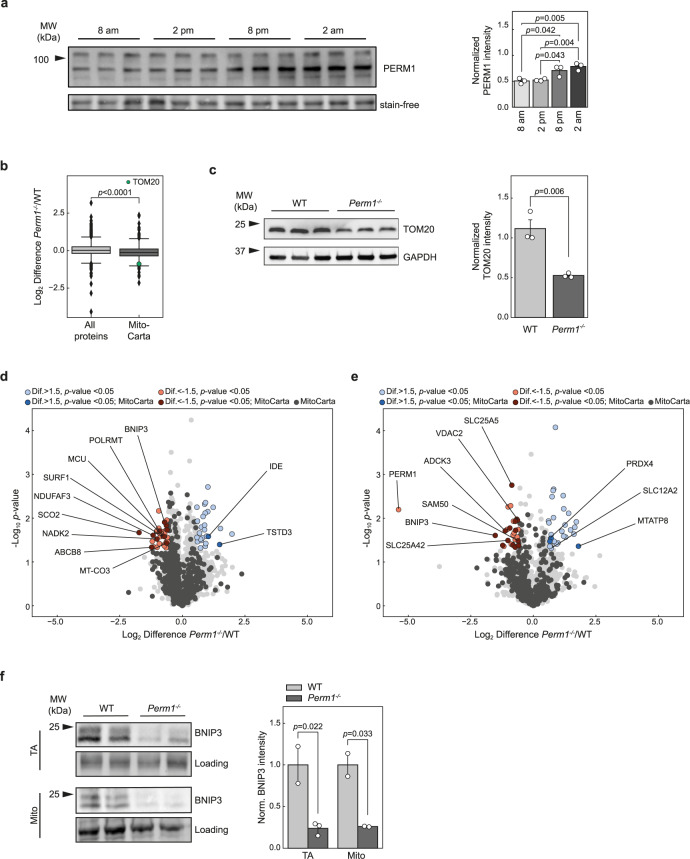
Inactivation of *Perm1* affects mitochondrial proteins related to transportation and breakdown of ROS. **a** Protein levels of PERM1 at different timepoints in TA muscle determined by western blotting. Normalized protein levels are expressed relative to the mean levels of each protein in the stain-free control (*n* = 3 mice per time point). **b** Comparison of the log_2_ differences in MitoCarta 2.0 proteins compared to the complete dataset revealed overall downregulation of mitochondrial proteins in TA muscle isolated from *Perm1*^*−/−*^ mice compared to wild-type controls (*n* = 2 mice per group). **c** Western blot and quantification of TOM20 in *Perm1*^*−/−*^ TA muscle and wild-type controls, substantiating the reduced mitochondrial content (*n* = 3 mice per group). **d**, **e** Volcano plots depicting significantly regulated proteins in *Perm1*^*−/−*^ TA muscle (**d**) and isolated TA muscle mitochondria (**e**) at 11 pm compared to wild-type controls (*n* = 2 mice per group). Cut-off was defined as *p* < 0.05 with a fold change >1.5. Darker colors indicate the MitoCarta 2.0 annotations. **f** Western blot and quantification, depicting downregulation of BNIP3 in TA muscle and isolated TA muscle mitochondria from *Perm1*^*−/−*^ mice. BNIP3 levels are expressed relative to the mean levels in wild-type control samples (*n* = 2 mice per group). Box plot (**b**) represents the median, 25th, and 75th percentiles, maximum and minimum are connected through whiskers. Outliers are defined as Q_1_–1.8 IQR and Q_3_+1.8 IQR. **a**, **d**, **e** Unpaired two-sided Student’s *t* test, S_0_=0.1, (**c**) unpaired two-sided Student’s *t* test, (**b**) unpaired two-sided Mann–Whitney *U*-test. Source data are provided as Source Data file and in Supplementary Data 2.

In a previous quantitative proteomic analysis of *Perm1*^*−/−*^ heart tissue, we showed that PERM1 has a stronger effect on protein expression during nocturnal activities than under daytime resting conditions^14^. To quantify circadian PERM1 protein expression, we isolated skeletal muscle tissues from wild-type mice every six hours and performed immunoblot analysis. Densitometric analysis revealed upregulation of PERM1 during the night when the physical activity of mice is at its peak (Fig. 3a).

To identify proteins that directly depend on PERM1, we measured the proteome of muscles during active nighttime and during daytime when PERM1 levels decrease. In total, we quantified 4686 proteins and observed 129 significantly regulated proteins (Fig. 3b–e, SI Fig. 2b–f; Supplementary Data 2); 18% of the quantified proteins in the nighttime proteomic dataset represent mitochondrial proteins. Interestingly, the overall fold-changes in mitochondrial proteins were significantly lower in *Perm1*^*−/−*^ mice compared to wild-type controls (Fig. 3b), while the fold-changes for all proteins were similar between the genotypes. This result was substantiated by immunoblotting with the mitochondrial marker protein TOM20 (Fig. 3c). Collectively, this finding suggests an overall reduction in the mitochondrial content of *Perm1*^*−/−*^ skeletal muscle, which is in line with the reduction of mtDNA. Downregulated mitochondrial proteins included proteins associated with OXPHOS function and OXPHOS assembly, namely SCO2 and NDUFAF3, surfeit locus protein 1 (SURF1), and cytochrome *c* oxidase subunit 3 (COX3; Fig. 3d).

Next, we isolated mitochondria from *Perm1*^*−/−*^ and wild-type TA muscles and mass spectrometric analysis identified 2884 proteins, including 453 mitochondrial proteins (Fig. 3e, Supplementary Data 2). We observed 58 significantly regulated proteins, of which 28 were increased and 30 decreased. Knockdown of *Perm1* in C2C12 cells downregulated mRNA expression of the OMM carnitine palmitoyltransferase CPT1B after PGC-1α induction^12^. Similarly, we observed reduced CPT1B protein expression in *Perm1*^*−/−*^ mitochondria during the night, suggesting attenuated transport of acylcarnitine.

Another essential transport protein, sorting and assembly factor SAM50 is a member of the MIB complex involved in sorting beta-barrel proteins at the OMM after import by the TOM complex. We observed a 1.5-fold reduction (*p*-value 0.03) in SAM50 in *Perm1*^*−/−*^ mitochondria compared to controls. In addition, FAM120A, another protein downregulated in *Perm1*^*−/−*^ mitochondria, is related to oxidative stress signaling and has been linked to the family of constitutive coactivators of the PPARγ family due to its sequence similarity. A recent study that named FAM120 oxidative stress-associated Src activator (OSSA) reported the protein functions as an activator of PI3K-AKT signaling and antiapoptotic pathways^25^. PERM1 is required for activation of selective PGC-1α target genes. Depletion of *Perm1* in PGC-1α-stimulated cells reduced the expression of several genes related to mitochondrial energy metabolism, including the DNA-directed RNA polymerase POLRMT and mitochondrial dimethyladenosine transferase 1 and 2 (TFB1M, TFB2M)^13^. Accordingly, we observed significant downregulation of POLRMT in *Perm1*^*−/−*^ skeletal muscle tissue during the night. In contrast, the mitochondrial replication factor TFAM and POLRMT interactors TFB1M and TFB2M were not altered in *Perm1*^*−/−*^ mutants. Enhanced degradation of mitochondria could also reduce the amount of mtDNA and mitochondrial proteins. However, markers of mitochondrial fusion/fission and mitophagy, such as mitofusin-1/2, OPA1, sequestosome (p62), and several autophagy-related proteins (ATG5, ATG7, ATGl1) were not altered in *Perm1*^*−/−*^ mutants (Supplementary Data 2). Additionally, the apoptosis-inducing protein BNIP3 was severely reduced in both *Perm1*^*−/−*^ whole muscle tissues and isolated mitochondria^26^. We substantiated the downregulation of BNIP3 by immunoblotting (Fig. 3f).

Besides diurnal changes of PERM1, we were also interested in PERM1 regulation during aging. We could not observe obvious changes in PERM1 protein expression levels between 3-month-old (young) and 24-month-old (aged) in TA and soleus muscles of wild-type mice (SI Fig. S2f). However, comparison of TA and soleus muscles of 2-year-old *Perm1*^*−/−*^ mutants and aged-matched wild-type control animals (*n* = 3; SI Fig. S2b, c) revealed significant differences in expression of 60 out of 3522 proteins, including 17 mitochondrial proteins (Supplementary Data 2), including prohibitin (PHB) and SIRT3, which were decreased in aged *Perm1*^*−/−*^ muscles.

In summary, ablation of *Perm1* leads to dysregulation of several proteins related to membrane transport and inactivation of reactive oxygen species (ROS). Since mitochondria are the main source of oxidative stress, this dysregulation could lead to cellular damage and reduced mitochondrial function. However, it remains unclear whether ablation of *Perm1* directly regulates the expression of these proteins or reflects a secondary adaptation.

### *Perm1*-deficient muscles exhibit reduced OXPHOS activity and higher turnover of mitochondrial proteins

**Fig. 4:**
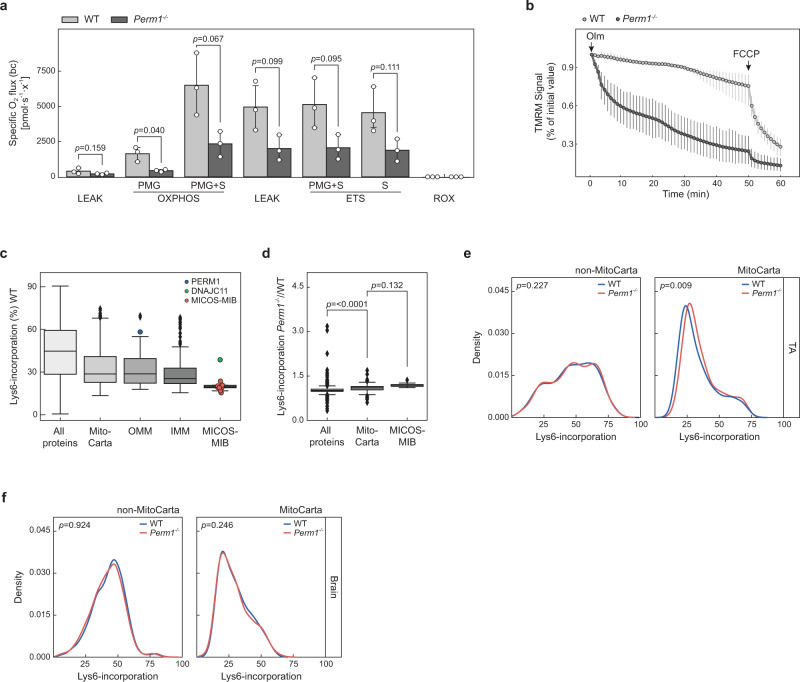
*Perm1*-deficient muscles show reduced oxygen flux and faster turnover of mitochondrial proteins. **a** High-resolution respirometry of isolated muscle mitochondria showing decreased specific oxygen flux in isolated *Perm1*^*−/−*^ mitochondria compared to wild-type controls (*n* = 3 mice per group). **b** FDB muscle fibers were loaded with 2.5 nM TMRM. Arrows indicate the addition of 5 µM oligomycin and 4 µM FCCP (*n* = 10 fibers in wild-type, *n* = 20 fibers in *Perm1*^*−/−*^). **c** Comparison of the Lys6-incorporation rates of all proteins, mitochondrial proteins, OMM proteins, IMM proteins, and proteins of the MICOS–MIB complex in wild-type mice fed a SILAC diet for 14 days (*n* = 3 mice per group). DNAJC11 and PERM1 had higher incorporation rates than other MICOS–MIB proteins. **d** Ratios of *Perm1*^*−/−*^ to wild-type control Lys6-incorporation rates for MitoCarta 2.0 proteins and MICOS–MIB proteins in comparison to the complete TA muscle dataset after feeding with a SILAC diet for 14 days. Enhanced labeling of mitochondrial proteins and MICOS–MIB proteins was observed in *Perm1*^*−/−*^ mice (*n* = 3 mice per group). **e**, **f** Density plots of the ratios of *Perm1*^*−/−*^ to wild-type control Lys6-incorporation for non-MitoCarta 2.0 and MitoCarta 2.0-annotated proteins, showing enhanced labeling of mitochondrial proteins in *Perm1*^*−/−*^ (**e**) TA but not in (**f**) *Perm1*^*−/−*^ brain. Data (**a**) are presented as mean values ± 0.95 CI. Box plots (**c**, **d**) represent the median, 25th, and 75th percentiles, maximum and minimum are connected through whiskers. Outliers are defined as Q_1_–1.8 IQR and Q_3_+1.8 IQR. (**a**) paired two-sided Student’s *t* test, (**d**, **e**, **f**) unpaired two-sided Mann-Whitney *U*-test. Source data are provided as Source Data file and in Supplementary Data 3. PMG (pyruvate, malate, glutamate), S (succinate), ETS (electron transfer system), ROX (residual oxygen consumption), Olm (oligomycin).

Since muscle strength data and proteomic analyses indicated reduced mitochondrial function, we examined the activity of the electron transport chain in mitochondria isolated from a mixture of several mouse leg muscles by high-resolution respirometry (Oroboros, O2k). Notably, oxygen flux was lower at all stages in *Perm1*^*−/−*^ samples compared to the wild-type controls (*n* = 3; Fig. 4a).

In addition to impairing oxidative phosphorylation, imbalanced mitochondrial metabolism may induce rapid depolarization of the inner mitochondrial membrane potential. Therefore, we isolated single muscle fibers from *Perm1*^*−/−*^ flexor digitorum brevis (FDB) and quantified mitochondrial membrane potential. Mitochondria from *Perm1*^*−/−*^ fibers showed higher depolarization when ATP synthase was inhibited by oligomycin, indicating that *Perm1*^*−/−*^ fibers are partially dependent on the reverse activity of ATP synthase to maintain membrane potential due to proton leakage-consuming proton-motive force (Fig. 4b).

To explore whether the turnover of mitochondria is affected, we used pulsed-SILAC labeling of living animals to monitor newly synthesized proteins via incorporation of the isotope-labeled amino acid ^13^C_6_-lysine (Lys6) by LC-MS/MS. A diet containing Lys6 was administrated to *Perm1*^*−/−*^ and wild-type control littermates for 14 days (*n* = 3). Skeletal muscle tissue was isolated and subjected to LC-MS/MS analysis to measure incorporation of stable isotopes (Supplementary Data 3). Brain tissue, which does not express *Perm1*, from the same animals was used as a control. In accordance with previous labeling studies^27^, we observed a Lys6-labeling rate of 29 ± 14% for mitochondria, substantially lower than the total proteome rate of 45 ± 18% (Fig. 4c). Interestingly, MICOS–MIB complex members had even lower Lys6-labeling rates of 20 ± 6% and thus are among the mitochondrial proteins with the lowest turnover. Conversely, PERM1 had a high Lys6-labeling rate of 58% (Fig. 4c, blue circle), followed by DNAJC11 (39%; Fig. 4c, green circle). We observed similar patterns by calculating the half-lives of these proteins in a dataset of soleus muscles of mice fed with a Lys6 diet for 1–4 weeks (S1 Fig. S3a; Supplementary Data 5). This finding might indicate that PERM1 and DNAJC11 have distinct stability and function compared to other OMM proteins or MICOS–MIB complex members. Similarly, OXPHOS complex assembly factors showed comparably high Lys6-labeling rates of 48 ± 12%.

Next, we compared the Lys6-incorporation rates of *Perm1*^*−/−*^ and wild-type controls. No differences in Lys6-incorporation rates were observed in brain tissue (SI Fig. S3b). In contrast, analysis of skeletal muscle tissue revealed enhanced Lys6-incorporation rates for *Perm1*^*−/−*^ mitochondrial proteins compared to controls. This effect was even more pronounced for MICOS–MIB complex members (Fig. 4d). Selected MS spectra of MIC60 and SAM50 are shown in SI Fig. S3c, d. The density and scatter plots illustrate the shift to higher Lys6-incorporation rates in the TA muscle, but not brain, of *Perm1*^−/−^ mutants compared to wild-type controls (Fig. 4e, f, SI Fig. S3e, h). This effect was most pronounced for MIC13, with a ~1.4-fold higher Lys6-labeling in *Perm1*^−/−^ mutants than wild-type (SI Fig. 3e, f). We concluded that *Perm1*^*−/−*^ mitochondria, particularly MICOS–MIB members, have a higher turnover compared to mitochondria from wild-type control muscles. Overall, loss of PERM1 significantly impaired oxidative phosphorylation and enhanced membrane depolarization, which may increase protein synthesis, especially of MIB complex members.

### PERM1 interacts with components of the mitochondrial intermembrane bridging complex

**Fig. 5:**
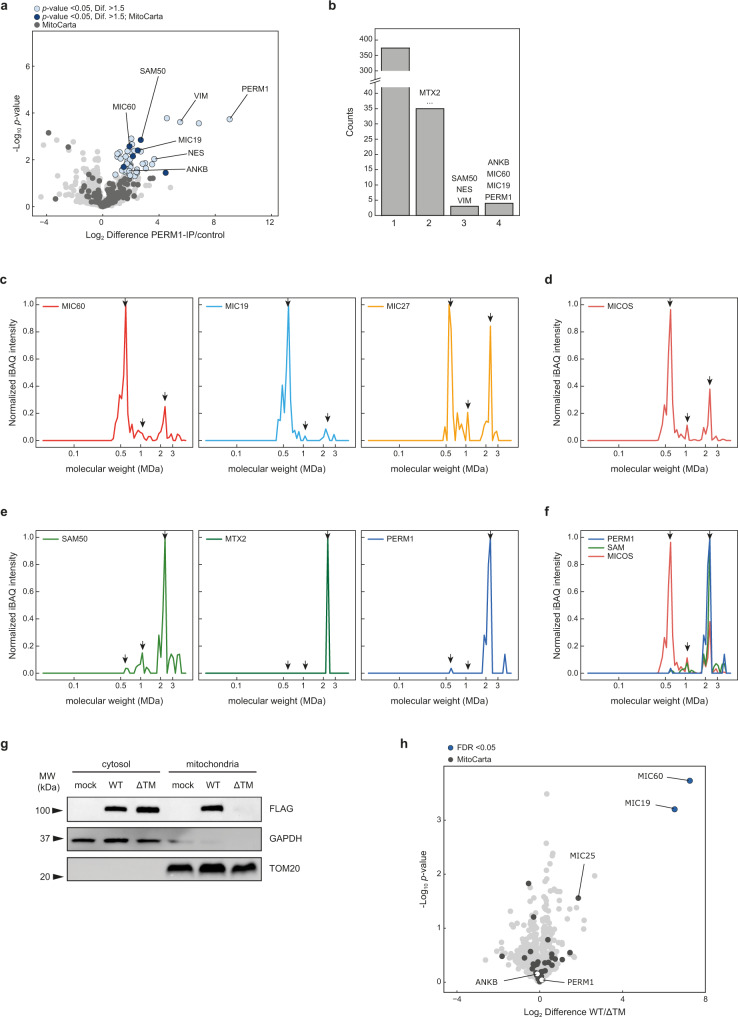
PERM1 interacts with components of the mitochondrial intermembrane bridging complex. **a** Volcano plot depicting significantly enriched proteins after immunoprecipitation of endogenous PERM1 in TA muscle (*n* = 3 mice per group). Cut-off was set to *p* < 0.05 with a fold change >1.5. Dark circles indicate MitoCarta 2.0 annotation. **b** Bar diagram showing the number of significantly enriched proteins (same cut-off as in volcano plot) identified in one, two, three, or all four immunoprecipitation approaches highlighting the most frequently found proteins. **c**–**f** Complexome profiling of muscle mitochondria; iBAQ-values were maximum normalized. Arrows indicate the three main complex assemblies. **c** Single profiles of the MICOS complex proteins MIC60, MIC19, and MIC27. **d** Summary plot of all MICOS complex members detected (MIC10, MIC19, MIC26, MIC27, and MIC60). **e** Single profiles of the SAM complex proteins SAM50 and MTX2, as well PERM1. **f** Summary plot of all detected MICOS complex members (red line), SAM components (green line; SAM50, MTX2), and PERM1 (blue line). **g** Deletion of the transmembrane helix in PERM1 resulted in loss of PERM1 in the mitochondrial fraction, as visualized by immunoblotting of cytosolic and mitochondrial fractions obtained from HEK-293T cells transfected with FLAG-tagged wild-type *Perm1* and FLAG-tagged ΔTM mutant. **h** Volcano plot depicting significantly enriched proteins after immunoprecipitation of PERM1 in HEK-293T cells transfected with FLAG-tagged wild-type *Perm1* and FLAG-tagged ΔTM mutant (*n* = 3 samples per group). The cut-off was an FDR < 0.05; dark circles indicate MitoCarta 2.0 annotations. MICOS proteins MIC60 and MIC19 were only found in wild-type immunoprecipitates, while ANKB and PERM1 were similarly enriched in wild-type and ΔTM immunoprecipitates. **a** unpaired two-sided Student’s *t* test, *S*_0_ = 0.1, **h** unpaired two-sided Student’s *t* test, *S*_0_=0.1, permutation-based FDR=0.05, 500 randomizations. Source data are provided as Source Data file and in Supplementary Data 4.

To clarify which proteins interact with PERM1, we performed affinity enrichment mass spectrometry in TREx-293 cells stably overexpressing *Perm1*-FLAG and C2C12 cells transiently overexpressing *Perm1*-FLAG. A PERM1 antibody was employed to identify endogenous PERM1 interactors in skeletal muscle and heart (Supplementary Data 4, Fig. 5a, SI Fig. S4a–c). A volcano plot was used to visualize PERM1 interactors (Fig. 5a) and the frequency with which these candidates were found is shown in Fig. 5b. Interestingly, we found that PERM1 interacts with several MICOS–MIB complex members, including MIC60, MIC19, SAM50 and MTX2. The interaction of PERM1 with SLC25A5 might explain the reduced protein abundance of this transporter in *Perm1*^−/−^ mitochondria (SI Fig. S4a, b). Detection of ankyrin B (ANKB or ANK2) and the intermediate filament proteins vimentin and nestin suggest that PERM1 connects the OMM to cytoskeletal structures. Reverse immunoprecipitations confirmed that MIC60 and MIC19 interact with PERM1 (SI Fig. S4d). Immunoblotting revealed similar levels of PERM1, MIC60 and MTX2 expression between different muscle groups, suggesting a similar structure of the MICOS–MIB complex (SI Fig. S4e).

To further investigate the interaction between PERM1 and the MICOS–MIB complex, we performed complexome profiling of mitochondria isolated from skeletal muscle by blue-native gel electrophoresis (BN-PAGE) and mass spectrometry. Each BN-PAGE lane was sliced into 70 sections and subjected to MS analysis. Intensity-Based Absolute Quantification (iBAQ) was used to quantify the proteins. All protein intensities were normalized and the profiles of selected MICOS (Fig. 5c, d, SI Fig. S4f) and MIB (Fig. 5e, f) complex members were plotted^28^. To assign protein intensities according to their size in Dalton (Da), respiratory chain complex standards of known sizes were used for normalization. The MICOS–MIB complex appears in different forms, depending on its maturation stage: (i) the 500–700 kDa IMM/intermembrane space (IMS) complex, which contains all MICOS subunits; (ii) the 800 and 1400 kDa SAM complex (SAM50, MTX1 + 2) that associates with the OMM; and (iii) the mature MICOS–MIB complex with a molecular weight between 2.2 and 2.8 MDa.

The 85 kDa protein PERM1 was mainly detected at a molecular weight of ~2.2 MDa in isolated mitochondria from muscle tissue (Fig. 5e). The core MICOS complex protein MIC60 was identified in all three maturing forms of the complex, whereas the MIB complex proteins SAM50 and MTX2 had similar migration profiles to PERM1 with a signal at ~2.2 MDa. Thus, we conclude PERM1 is a member of the mature MICOS–MIB complex in skeletal muscle tissue.

### PERM1 anchors to the OMM via a conserved transmembrane helix

Next, we examined the PERM1 amino acid sequence to identify domains that potentially interact with proteins of the MICOS–MIB complex. Although most of the PERM1 protein is intrinsically disordered, a secondary structure and topology prediction of all-helix integral membrane proteins using MEMSAT3^29^ predicted a hydrophobic transmembrane helix from amino acids 740–755 at the carboxyl-terminus of PERM1, which is followed by a 52 amino acids long tail. Furthermore, the C-terminal segment (732–803) shows high sequence conservation between human, mouse and bovine PERM1, while the overall amino acid sequence identity is lower than 50%. This may indicate that PERM1 anchors into the outer mitochondrial membrane via its C-terminal domain.

To test this hypothesis, we deleted amino acids 736–759 in the PERM1-FLAG construct. Strikingly, separation of the cytosolic and mitochondrial fractions of HEK-293T cells transfected with the mutant PERM1 (hereafter referred to as ΔTM mutant) resulted in the loss of PERM1 in the mitochondrial fractions, while overexpressed wild-type PERM1 was present in both mitochondrial and cytosolic fractions (Fig. 5g). Furthermore, deletion of the hydrophobic transmembrane helix prevented interactions between PERM1 and MIC60, MIC19, and MIC25 in immunoprecipitation experiments (Fig. 5h, Supplementary Data 4). In contrast, no differences were observed in the interaction of ΔTM PERM1 with ANKB compared to wild-type PERM1. We concluded that the C-terminal helical structure is responsible for mitochondrial localization of PERM1 and its interaction with the MICOS–MIB complex.

### Altered localization of the adaptor protein ankyrin B in *Perm1*^*−/−*^ muscles

**Fig. 6:**
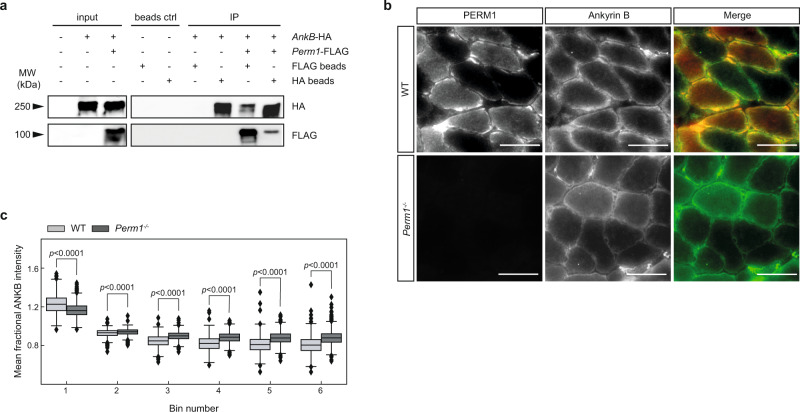
PERM1 associates with ankyrin B and the interaction with the MICOS–MIB complex is dependent on the transmembrane helix. **a** PERM1 interacts with ANKB, as indicated by immunoprecipitation of FLAG-tagged *Perm1* and HA-tagged ankyrin-B in TREx-293 cells stably expressing *Perm1*-FLAG. Protein lysates were immunoprecipitated with anti-FLAG beads and anti-HA beads and immunoblotted with anti-FLAG and anti-HA antibodies. **b** Immunohistochemical staining of soleus muscle revealed co-localization of PERM1 (red) with ANKB (green). Scale, 50 µm. **c** Mean fractional intensity of ANKB signals in bins 1–6 from *Perm1*^*−/−*^ and wild-type soleus muscle fibers. ANKB staining intensity was reduced at subsarcolemmal sites and increased within interfibrillar mitochondria in *Perm1*^*−/−*^ muscles compared to wild-type controls (*n* = 4 mice per group, 2299 quantified fibers). Box plot (c) represents the median, 25th, and 75th percentiles, maximum and minimum are connected through whiskers. Outliers are defined as Q_1_–1.8 IQR and Q_3_+1.8 IQR. (c) unpaired two-sided Mann-Whitney *U*-test. Source data are provided as Source Data file.

We found that PERM1 does not only interact with members of the MICOS–MIB complex, but also with several proteins associated with membrane and cytoskeletal structures including ANKB, an adaptor protein that interacts with the cytoskeleton and connects transmembrane proteins with the sarcolemma^30^ (Fig. 5a). The PERM1-ANKB interaction was verified by co-immunoprecipitating FLAG-tagged *Perm1* and HA-tagged *AnkB* in TREx-293 cells (Fig. 6a). Immunostaining confirmed co-localization of PERM1 and ANKB in the periphery of soleus muscle fibers (Fig. 6b). Interestingly, we also observed a reduction in the ANKB signals at the periphery of *Perm1*^*−/−*^ mutant muscles. This finding was similar to the results of immunostaining with the TOM20 antibody (Fig. 1j). To quantify the reduction in ANKB, we separated each fiber into six bins (as shown in Fig. 1i) and measured the intensity of ANKB in *Perm1*^*−/−*^ and wild-type muscle sections (*n* = 4). ANKB signal intensity was significantly lower at subsarcolemmal sites and higher within interfibrillar regions in *Perm1*^*−/−*^ mutants (Fig. 6c, 2299 quantified fibers). These results validate the interaction of PERM1 with ANKB and suggest this interaction could be responsible for the localization of mitochondria at the sarcolemma.

## Discussion

We here demonstrate that PERM1 interacts with the MICOS–MIB complex in mitochondria and with the intracellular adaptor protein ankyrin B to tether a subpopulation of skeletal muscle mitochondria to the sarcolemma. We show that PERM1 is required for mitochondria, providing the energy to achieve full muscle strength. So far, detailed knowledge about the physiological role of PERM1 was missing. PERM1, which rapidly upregulated when human skeletal muscle tissue becomes physically active, was recently described as a tissue-specific regulator of oxidative capacity in cultured muscle cells^12, 31, 32^. In addition, a previous study suggested that PERM1 is required for expression of PGC-1α/ERR target genes in C2C12 myotubes, for PGC-1α-induced mitochondrial biogenesis, and maximal oxidative capacity during physical activity^13^. Although PERM1 was reported to localize to the cytosol and the nucleus, we recently showed that PERM1 associates with the OMM^14^, suggesting that PERM1 might shuttle between mitochondria and the nucleus. Quantitative interactome screenings demonstrated that PERM1 physically interacts with MICOS–MIB subunits, which was confirmed by BN-PAGE analysis indicating that PERM1 co-migrates with the mature MICOS–MIB complex (Fig. 5). We hypothesize that the C-terminal transmembrane helix anchors PERM1 in the OMM, while the most C-terminal, 52 amino acid sequence, may extend into the IMS to allow interaction with MIC60 and MIC19. Further experiments are required to investigate the mechanisms that integrate PERM1 into the membrane and to answer the question whether PERM1 is involved in mitochondrial transport processes.

Previous studies demonstrated that SSM and IFM have distinct biochemical properties, capacities for respiration, and sensitivities to metabolic stress. Since PERM1 is enriched in SSM and loss of PERM1 results in a strong reduction in SSM, we hypothesize that PERM1 is critical for the specific functions of SSM and IFM. Several mitochondrial inner and outer membrane transporters were downregulated in *Perm1* mutant skeletal muscles. We assume that PERM1 stabilizes the MICOS–MIB complex and prevents degradation of its subunits, since in vivo labeling with the stable isotope Lys6 demonstrated increased synthesis of several MICOS–MIB members including SAM50, MTX2 and MIC13 in *Perm1*^*−/−*^ mutants (Fig. 4f).

Circadian rhythm regulates numerous transcriptional and translational processes that influence cellular metabolism and mitochondrial activity. We recently showed that CK2-dependent phosphorylation promotes rapid breakdown of PERM1 by ubiquitination. Interestingly, CK2 is involved in the control of the mammalian circadian clock, since inhibition of CK2 activity decreases the amplitude and increases the period of circadian gene expression oscillations^33^. Moreover, the activity of CK2 regulation of the carbohydrate metabolism is modulated by insulin^34^, indicating a central role for CK2 in regulating insulin-sensitivity, energy homeostasis and tissue remodeling. In contrast to CK2, which is upregulated in skeletal muscle of patients with diabetes mellitus type 2, PERM1 is decreased, further supporting a close association with insulin-dependent metabolism and mitochondrial activity^35^. The lower CK2 levels during the night, when mice are more active, coincide with reduced phosphorylation and degradation of PERM1, which increases mitochondrial activity. Hence, it is tempting to speculate that CK2-dependent PERM1 phosphorylation determines the circadian rhythm of PERM1 and thereby contributes to the circadian regulation of mitochondrial activity. We recently described more than 30 phosphorylation sites on PERM1 in cardiomyocytes. Thus, it seems likely that in addition to CK2 other kinases, including PKC, CDC2 and p38, phosphorylate and regulate PERM1. Phosphoproteomics profiling of *Perm1*^*−/−*^ and wild-type muscles under sedentary and activated conditions might help to decipher the mechanisms by which kinases regulate PERM1 and mitochondrial activity. In particular, analysis of mouse models of insulin resistance, such as the *db/db* mouse or administration of high caloric diets, may shed further light on this interplay.

Our data suggest that PERM1 is not a central structural component of the MICOS–MIB complex, but rather functions as a modulator of its activity or a sensor for energy requirements. The formation of cristae junctions and contact sites is a dynamic process that, depending on energy demands, involves continuous remodeling. The changes in PERM1 protein expression, its short half-life of ~4 days (SI Fig. S3a), and rapid degradation by CK2-dependent phosphorylation of the PEST domain^14^ might potentially contribute to remodeling processes in mitochondria and thereby control mitochondrial energy production.

Interestingly, we observed a reduction in oxygen flow in all measured stages and mitochondrial dysfunction in *Perm1*-deficient muscles as indicated by enhanced mitochondrial membrane depolarization while no signs of apoptosis and mitophagy were apparent. In fact, we detected a strong downregulation of the proapoptotic factor BNIP3 and a reduction of the voltage-dependent anion channel VDAC2, which is part of the mitochondrial permeability transition pore facilitating cytochrome *c* release and induction of apoptosis. These observations make it not only unlikely that removal of SSM in *Perm1*^*−/−*^ muscles is mediated by mitophagy, but also suggest that absence of *Perm1*, together with the loss of BNIP3, leads to accumulation of damaged mitochondria. Genetic inactivation of *Bnip3* results in changes in the mitochondrial membrane potential and reduced oxygen consumption in the liver^36^, which was also seen in *Perm1*^−/−^ mutants.

In *Drosophila*, the MICOS component MIC60 controls mitochondrial motility by regulating protein levels of the OMM protein and Rho GTPase Miro, which anchors mitochondria to microtubule motors^37^. Mammalian skeletal muscle mitochondria display a similar subcellular distribution as the microtubular network and there is a close connection between the structural organization of mitochondria and energy flux^38^. Mitochondria accumulate at the periphery of muscles, and in particular, close to capillaries^39^, which reduces the distance for the diffusion of oxygen and metabolites from the capillaries to mitochondria. Moreover, SSM are thought to be primarily involved in the generation of the mitochondrial proton-motive force near capillaries, whereas IFM, which need to be close to myofibrils for efficient supply of ATP or other high-energy phosphates, use the generated proton-motive force to produce ATP^4^. Disruption of the finely balanced mitochondrial network by removal of components linking mitochondria to the microtubular and intermediate filament networks or breakdown of the intermediate filament network, will disrupt division of labor within mitochondrial subpopulations and reduce muscle strength^38^.

Our protein-protein interaction screening also identified the intermediate filament proteins vimentin and nestin, as well as ankyrin B (ANKB) as potential interactors of PERM1 (Fig. 3a). Vimentin co-localizes with mitochondria, regulates mitochondrial distribution, and influences the rate of ATP synthesis and mitochondrial membrane potential^40^. Heteropolymeric intermediate filaments consisting of nestin and vimentin are responsible for the connection of myofibrillary Z-lines via interactions with α-actinin to ANKB-containing costameres at sarcolemma^41, 42^. ANKB is one of three members of the ankyrin family of plasma membrane adaptor proteins involved in the organization and anchoring of integral membrane complexes to the spectrin- and actin-based cytoskeleton. Moreover, ANKB functions as an adaptor protein for dystrophin and dynactin and ankyrin-dependent organization of those proteins are required to maintain sarcolemmal integrity during physical exercise^43^. Loss of ANKB leads to the intracellular distribution of dystrophin and destabilization of costamere-associated microtubules and muscle fragility^42^. As ANKB also exhibits an intracellular localization^44^ and regulates the axonal transport of several organelles, including mitochondria, ANKB is assumed to serve as a core protein involved in intracellular organelle transport^45^. We demonstrated that ANKB co-localizes with PERM1 and mitochondria at the sarcolemma of skeletal muscles. Interestingly, *Perm1* ablation led to a reduction in ANKB at sarcolemma (Fig. 6). We reason that PERM1 and ANKB mediate the anchorage of the mitochondria-associated cytoskeleton to the sarcolemma, albeit it seems also possible that impaired mitochondrial transport along microtubules is responsible for the loss of SSM in *Perm1*^*−/−*^ mutants.

The characterized PERM1-ANKB interaction may reflect a novel mechanism of how mitochondria are recruited and connected to the sarcolemma in skeletal muscle tissue. Numerous structural and functional interactions have been associated with the integration of the mitochondria and the cytoskeleton, which influences the localization and modulates the activity and turnover of mitochondria. The dual interaction of PERM1 with the MICOS–MIB complex and intermediate filaments represents an unknown mechanism of how mitochondria are connected to the cytoskeleton and indicates that PERM1 is instrumental for the formation of the spatially organized, functional mitochondrial network that makes skeletal muscles strong.

## Methods

### Antibodies

The antibodies used for immunoblotting, immunoprecipitation and immunohistochemistry were: PERM1 (Sigma, #HPA031711), diluted 1:1,000 for immunoblotting, 1:100 for immunohistochemistry; BNIP3 (Cell Signaling, #3769), diluted 1:1,000; TOM20 (Abcam, #ab56783), diluted 1:100 for immunohistochemistry; TOM20 (Sigma, #HPA011562), diluted 1:5,000 for immunoblotting, 1:100 for immunohistochemistry; GAPDH (Invitrogen, #AM4300), diluted 1:10,000 for immunoblotting; Ankyrin B (Invitrogen, #33-3700), diluted 1:1000 for immunoblotting, 1:100 for immunohistochemistry; MYH7 (Developmental Studies Hybridoma Bank [DSHB], BA-F8), MYH2 (DSHB, SC-71), MYH4 (DSHB, BF-F3), CD31/PECAM (BD Pharmingen, #553370), all diluted 1:100 for immunohistochemistry; anti-FLAG M2-HRP (Sigma, #A8592), anti-HA-HRP (Miltenyi, #130-091-972), Alexa Fluor 350 anti-mouse IgG2b (Life Technologies, #A-21140), Alexa Fluor 488 anti-mouse IgG1 (Life Technologies, #A-21121), Alexa Fluor 546 anti-mouse IgM (Life Technologies, #A-21045), Alexa Fluor 546 anti-rabbit (H+L) (Life Technologies, #A-11010), Alexa Fluor 488 anti-rat (Life Technologies, #A-11006), anti-mouse HRP (Sigma, #A9044) and anti-rabbit HRP (Sigma, #A0545), all diluted 1:4,000 for immunoblotting and 1:200 for immunohistochemistry.

### Generation of Perm1^−/−^ mice

*Perm1*^*−/−*^ mice were generated in the mouse facility of the Max-Planck-Institute in Bad Nauheim and transferred to the CECAD mouse facility via embryo transfer, as previously described^14^. All animal experiments were performed in accordance with national and institutional guidelines and procedures were approved by the responsible local authorities in Germany (Landesamt für Natur, Umwelt und Verbraucherschutz Nordrhein-Westfalen, Germany). Mice were housed at 22 ± 2 °C, with a humidity of 55 ± 10%, and an air exchange rate of 15 times per hour on a 12 h light-dark cycle with free access to food and water.

### Plasmids

The mouse PERM1 coding sequence was cloned into the pcDNA5/TO vector containing a C-terminal FLAG tag, as previously described^14^. AnkyrinB-2XHA was a gift from Vann Bennett (Addgene plasmid #31057; http://n2t.net/addgene:31057; RRID: Addgene_31057)^42^.

To generate the transmembrane mutant, amino acids 736-759 were deleted from the *Perm1*-FLAG plasmid using the In-Fusion cloning technique (TaKaRa Bio Inc.) following the manufacturer’s instructions. In brief, 10 ng *Perm1*-FLAG plasmid was amplified using PrimeSTAR GXL DNA polymerase (TaKaRa Bio Inc.). The PCR product was isolated by gel extraction and incubated with DpnI (Thermo Fisher) at 37 °C for 1 h. The In-Fusion cloning reaction was performed as recommended, and the reaction mixture was transformed into competent DH5α cells. Primers are listed in Supplementary Table 1.

### Treadmill and weight experiments

Treadmill and weights experiments were performed in collaboration with the group of Prof. Dr. Bloch at German Sport University, Cologne. After the experiments, the mice were placed back in their cage and returned to the mouse room with free access to water and food.

### Treadmill running

Mice (*n* = 9) were placed individually on a treadmill (Columbus Instruments). The speed was started at 8 m/min and increased by 2 m/min every two minutes up to 18 m/min. Mice were gently placed back at the correct position if they stopped running. The session was terminated when the mouse met either of the exhaustion criteria: if the mouse was replaced on the treadmill for the third time or stopped running for more than 10 s.

### Inverted screen

The strength of the *Perm1*^*−/−*^ and wild-type mice was measured as previously described^46^. The inverted screen test was performed following the Kondziella protocol^47^. The screen is a 43 cm square of wire mesh consisting of 12 mm squares of 1 mm diameter with a 4 cm-deep wooden surround. Mice (*n* = 9) were placed individually in the center of the wire mesh and the screen was rotated to an inverted position and held steadily 40–50 cm above a padded surface. The time until the mouse could not hold on to the screen anymore was recorded; the test was terminated after 12 min.

### Weight test

Weight tests were conducted using a series of six weights ranging from 20 and 85 g; the lightest weight was offered first. The weights were attached to wires that can be grabbed by the claws of the mouse. The mice (*n* = 9) were held by the middle of the tail and lowered so they could grasp the weight on the laboratory bench. A maximal holding time of 3 s was allowed; if the mouse dropped the weight in less than 3 s, the time was recorded. If the weight was dropped, the mouse was rested for about 10 s and offered the same weight again; failing three times was the termination criteria.

### Skeletal muscle force measurements

Skeletal muscle force in vivo was determined as described previously^48^. Briefly, mice were anesthetized by intraperitoneal injection of xylazine (Xilor; 20 mg/kg) and Zoletil (10 mg/kg). Z small incision was made from the knee to the hip, to expose the sciatic nerve. Before the branch of the sciatic nerve, Teflon-coated 7 multi-stranded steel wires (AS 632; Cooner Sales, Chatsworth, CA, USA) were implanted with sutures on either side of the sciatic nerve. To avoid recruitment of the ankle dorsal flexors, the common peroneal nerve was cut. Torque production of the plantar flexors after nerve stimulation was measured using a muscle lever system (Model 305 C; Aurora Scientific, Aurora, ON, Canada). Normalized muscle force was determined by dividing absolute force by muscle mass.

### Cell culture

All cell lines were cultivated as an adherent fibroblastoid monolayers in 10 cm dishes in DMEM (Gibco) supplemented with 10% FBS and 1× Penicillin-Streptavidin-Glutamine mix at 37 °C and 5% CO_2_. Cells were washed once with ice-cold PBS, harvested in 500 µL RIPA buffer supplemented with protease and phosphatase inhibitors, incubated for 30 min at 4 °C with rotating, and DNA was sheared by sonification. Lysates were centrifuged at 18,000 *g* for 10 min and 4 °C to pellet cell debris.

### Immunohistochemistry

Cross-sections of soleus and TA (10 µm) were prepared by snap-freezing whole muscles in liquid nitrogen-cooled isopentane. Sections were fixed in −20 °C cold acetone for 8 min, blocked in 0.1% Triton, 5% goat serum and 2% BSA in TBS for 1 h at RT, incubated with the primary antibody in 1:10 blocking solution in a humidified chamber at 4 °C overnight, mounted with Aqua Poly mount (Polysciences) and imaged using a DMi8A inverse microscope (Leica).

Quantitative image analysis was done using the cell image analysis software CellProfiler™ (version 3.1.9)^49^ to quantify the intensity of the TOM20 and ANKB signals across the muscle fibers. Two pipelines were generated: The first pipeline was created to identify single muscle fibers in an entire muscle cross-section to generate a binary image mask for further analysis. To identify the fiber outlines, the cross-sections were additionally stained with antibodies against type I (BA-F8) and type IIa (SC-71) fibers. Here, a combination of the images of the type I and type IIa fiber staining and an enhanced image of the TOM20/ANKB staining was used for mask generation. In the second pipeline, the generated binary image mask was used to measure the object intensity distribution of the TOM20/ANKB staining in six scaled bins per muscle fiber. Fibers that were not correctly recognized with the image mask were manually excluded from the analysis. The mean fractional intensity for each bin calculated as the fraction of total intensity normalized by the fraction of pixels for each bin was used for statistical analysis (unpaired two-sided Mann-Whitney *U*-test).

### Pulsed SILAC labeling of Perm1^−/−^ mice

Time-dependent Lys6-incorporation assays were conducted to monitor protein synthesis in vivo. In the first study, three mice of each genotype (*Perm1*^*−/−*^ and wild-type control) were voluntarily fed a Lys6-containing SILAC diet for two weeks (3.0 g of ^13^C6-lysine containing diet; Silantes GmbH, Lys(6)-SILAC-Mouse Diet, purity > 97%). In the second study, wild-type mice were labeled for 7–28 days (*n* = 3 mice per time point) with the Lys6-containing SILAC diet. Several organs and tissues were prepared for LC/MS-MS analysis. Briefly, the tissues were lyophilized overnight, homogenized with a Precellys^TM^ tissue homogenizer, and a small amount of powder was lysed with modified RIPA buffer (100 mM Tris/HCl pH 7.5, 300 mM NaCl, 2% Nonidet P40, 2 mM EDTA, 0.2% sodium deoxycholate), and protein samples were subsequently in-gel digested with the protease LysC overnight to generate peptides that contain at least one lysine.

### Tissue lysis

Mouse tissues were snap frozen in liquid nitrogen, ground to a fine powder using a mortar and pestle, and resuspended in modified RIPA buffer (100 mM Tris/HCl pH 7.5, 300 mM NaCl, 2% Nonidet P40, 2 mM EDTA, 0.2% sodium deoxycholate) supplemented with protease and phosphatase inhibitors at a buffer to tissue ratio of 15:1 Lysates was then incubated with rotation for 30 min at 4 °C, sonicated for 10 min at 30 s intervals at 4 °C using a Bioruptor® Pico (Diagenode). Cell debris was pelleted by centrifugation at 18,000 *g* for 10 min at 4 °C.

### Protein concentration determination

Protein concentrations were determined using the Pierce Protein Assay (Thermo Fisher). BSA standard solutions (0.05 mg/mL to 1.0 mg/mL) were generated by diluting 2 mg/mL BSA solution (Thermo Fisher). Protein samples were diluted 1:10 in modified RIPA buffer (100 mM Tris/HCl pH 7.5, 300 mM NaCl, 2% Nonidet P40, 2 mM EDTA, 0.2% sodium deoxycholate). Absorption was measured at 660 nm using an EnSpire® Multimode Plate Reader (Perkin Elmer). To enable measurement of protein samples diluted in SDS-containing buffer, Pierce 660 nm Protein Assay Reagent was supplemented with Ionic Detergent Compatibility Reagent (Thermo Fisher) according to manufacturer’s instructions.

### Immunoprecipitations

To immunoprecipitate PERM1 in TREx-293 cells, cells stably expressing *Perm1*-FLAG or the control To5-STOP-FLAG vector were cultivated to 70-80% confluency as described above, treated with 10 µg/mL doxycycline for approximately 15 h, and lysed as described above. After removing cell debris, the supernatants were transferred into new precooled tubes and 40 µL anti-FLAG (Miltenyi) beads were added. The mixtures were incubated for 60 min at 4 °C with rotation and. separated using the magnetic µMACS system (Miltenyi) at 4 °C. The µColumns were equilibrated with lysis buffer, loaded with the lysate-bead-suspension, washed thrice with 200 µL lysis buffer, and washed thrice with 50 mM ammonium bicarbonate (ABC). Elution was performed at RT by applying 20 µL urea buffer (6 M Urea, 2 M Thiourea) for 10 min and collecting the eluate with 50 µL urea buffer. Finally, samples were prepared for MS analysis by in-solution/on-bead digestion.

To immunoprecipitate PERM1 in C2C12 cells, 6 × 10^5^ cells were seeded on day 1. On day 2, the cells were transfected with *Perm1*-FLAG or the control vector TO5-FLAG-STOP using GeneJuice^®^ Transfection Reagent (Novagen). Cells were harvested 24 h after transfection, as described above and immunoprecipitated as described for TREx-293 cells. To mimic the natural environment of PERM1 in the body, 100 µL of whole muscle and heart lysates prepared from wild-type mice were added with the anti-FLAG beads.

To immunoprecipitate endogenous PERM1 from tissue, 40 mg samples TA muscles and hearts from wild-type mice were lysed as described above. Protein concentrations were determined using Pierce Protein Assay. Two aliquots of 800 µg protein per sample were diluted to 500 µL with modified RIPA buffer (100 mM Tris/HCl pH 7.5, 300 mM NaCl, 2% Nonidet P40, 2 mM EDTA, 0.2% sodium deoxycholate): 40 µL Protein A beads and 0.9 µg PERM1 antibody was added to one aliquot; only 40 µL Protein A beads were added to the control sample. Immunoprecipitation steps were performed as described above.

To immunoprecipitate AnkB, cells stably expressing *Perm1*-FLAG were cultivated to 60-70% confluency prior to transfection with AnkyrinB-2XHA or control empty HA vector using the calcium phosphate method. In brief, 8 µg of DNA was incubated with 37 µg of 2 M CaCl_2_ and 300 µg 2× HBS (50 mM HEPES, 280 mM NaCl, 1.5 mM NaH_2_PO_4_, pH 7.0) for 30 min. Next, the mixture was added to the cultured cells and incubated for 24 h. After 9-10 h, the media was exchanged by fresh media containing 10 µg/mL doxycycline. Cells were harvested and lysed as described above. Immunoprecipitation was performed using the magnetic µMACS system (Miltenyi) as described above using anti-HA beads (Miltenyi).

Immunoprecipitation of the ΔTM mutant of PERM1 in HEK-293T cells was performed using the calcium phosphate transfection method, as described above.

### Isolation of mitochondria from tissue

Freshly collected heart or muscle samples were washed once in cold PBS, incubated on ice in 4 mL Mito buffer A (220 mM mannitol, 70 mM sucrose, 20 mM HEPES/KOH, 0.5 mM PMSF, 2 mg/mL BSA, 1 mM EDTA/Tris pH 7.4) supplemented with protease and phosphatase inhibitors, homogenized in a motor-driven potter (20 strokes, 1000 rpm) and cell debris was pelleted at 800 *g* for 5 min at 4 °C. The supernatants were collected, the pellet was homogenized again (20 strokes, 1000 rpm), centrifuged at 800 *g* for 5 min at 4 °C, the supernatant was combined with the supernatant from the first homogenization, and centrifuged at 8000 *g* for 5 min at 4 °C. The supernatant was discarded and the pellet was resuspended in 1 mL Mito buffer B (220 mM mannitol, 70 mM sucrose, 20 mM HEPES/KOH, 0.5 mM PMSF, 1 mM EDTA/Tris pH 7.4), centrifuged twice at 8000 *g* for 5 min at 4 °C and the mitochondrial pellet was resuspended in 1 mL buffer B. Finally, the samples were centrifuged at 10,000 *g* for 5 min at 4 °C and the mitochondria were resuspended in 300–500 µL Mito buffer B.

To investigate the localization of the ΔTM mutant, the cytosolic and mitochondrial fractions of cultured cells were separated, as described above. After homogenization at 8000 *g* and centrifugation, the resulting supernatant was designated as the cytosolic fraction and cleared twice by centrifugation at 8000 *g* for 5 min at 4 °C.

### Isolation of SSM and IFM

Dissected muscles from the mouse hind limb were washed in 2 mL of Isolation buffer (180 mM KCl, 10 mM EDTA, pH 7.4) on ice. After drying the tissue, it was weighed and cut into smaller pieces with sharp scissors. Depending on the muscle weight, the samples were supplied with four volumes of KEA buffer (180 mM KCl, 10 mM EDTA, 0.5% BSA pH 7.4) compared to the muscle weight (1 mg tissue/4 µl KEA) and homogenized in a motor-driven potter (10 strokes, 2000 rpm). Subsequently, after carefully transferring the homogenate to a tube, it was centrifuged at 100 *g* for 10 min. The supernatant containing the SSM was placed on ice in a new tube while the pellet was weighed and resuspended with two volumes of KEA buffer compared to the pellet weight. For the collection of the IFM, Nagase (Protease XXIV, Sigma P-8038, 0.1 mg/mL) was added in a 5 µl Nagase/1 mL resuspended pellet ratio for 35 sec with immediate centrifugation at 5900 *g* for 5 min. The supernatant was discarded, and the pellet was resuspended with one volume of KEA buffer and centrifuged at 100 *g* for 10 min. Afterwards, the supernatant containing the IFM and the SSM containing tube were centrifuged at 5900 *g* for 10 min. The pellets were washed three times with one volume of KME buffer (100 mM KCL, 0.5 mM EGTA, pH 7.4) and centrifuged at 5900 *g* for 10 min. After the final washing step, the SSM and IFM were resuspended in 0.5 volumes of KME buffer.

### In-gel digestion and sample purification

Proteomic samples of TA muscle tissue and isolated TA muscle mitochondria were prepared using a modified version of the in-gel digestion protocol by^50^. Briefly, lysates were precipitated overnight in four volumes (*v:v*) of ice-cold acetone. Protein pellets were extracted by centrifugation at 13,000 *g* for 10 min and dissolved in Bolt LDS sample buffer (Invitrogen). Dithiothreitol (DTT) was added to a final concentration of 10 mM. The mixtures were heated at 70 °C for 10 min, cooled to room temperature, iodoacetamide (IAA) was added to a final concentration of 55 mM and incubated in the dark for 20 min. Proteins were separated using Bolt 4–12% Bis–Tris Plus gels (Invitrogen), Coomassie-stained, and each lane was cut into six to ten slices. Each slice was chopped into 1 mm cubes, destained with 50% ACN and dehydrated with 100% ACN, digested with 3 ng/µL trypsin/LysC solution (90% trypsin/10% LysC) for 30 min at 4 °C, 50 mM ABC was added to the swollen gel pieces, incubated overnight at 37 °C and digestion was stopped by acidifying the samples with trifluoroacetic acid (TFA). Tryptic peptides were extracted with increasing concentrations of ACN and organic compounds were evaporated using a SpeedVac concentrator Plus (Eppendorf). Acidified samples were desalted using Stop and Go extraction tips^51^.

### In-solution/on-bead digestion

Eluates from immunoprecipitation experiments were prepared for MS analysis by in-solution/on-bead digestion. Urea-containing samples were reduced by applying DTT at a final concentration of 5 mM for 1 h at RT, alkylated with a final concentration of 40 mM IAA for 30 min in the dark at RT, digested with 1 µL LysC for 2-3 h at RT, diluted with 50 mM ABC to a urea concentration of 2 M, incubated with 1 µL 0.5 mg/mL trypsin overnight at RT, acidified to 1% FA and purified using StageTips.

### MS-based proteome analysis

Proteome samples were analyzed using a liquid chromatography tandem mass spectrometry set up in a Q-Exactive™ Plus Hybrid Quadrupole-Orbitrap™ (Thermo Fisher). Chromatographic peptide separation was achieved on PoroShell 120 packed 50 cm analytical columns coupled to an EASY-nLC 1000 HPLC system and a binary buffer system consisting of buffer A (0.1% FA) and buffer B (80% ACN, 0.1% FA). In-gel digested samples were analyzed over a 150 min gradient, raising the content of buffer B from 9 to 29% over 119 min, from 29 to 68% over 5 min, and from 68% to 95% over 16 min. The column was washed with 95% buffer B, buffer B was reduced to 5% over 5 min and the column was re-equilibrated with 5% buffer B for 5 min. Full MS spectra (300–1,750 *m/z)* were recorded at a resolution (R) of 70,000, maximum injection time (max. IT) of 20 ms and AGC target of 3e6. The ten most abundant ion peptides in each full MS scan were selected for HCD fragmentation at nominal collisional energy (NCE) of 25. MS2 spectra were recorded at R = 17,500, maximum IT of 60 ms, and AGC target of 5e5 (Xcalibur version 3.1).

Samples derived from immunoprecipitations and subsequent in-solution/on-bead digestion were measured over a 100-min gradient. The content of buffer B was increased from 13 to 48% within 75 min, increased to 75% over 5 min, washed with 95% buffer B for 10 min, reduced to 5% within 5 min and re-equilibrated with 5% buffer B for 5 min.

### MS data processing and analysis

Raw MS data were analyzed using MaxQuant analysis software and the implemented Andromeda software (1.5.3.8)^52, 53^. Peptides and proteins were identified using the mouse UniProt database with common contaminants (FASTA file downloaded on 16/6/2017, size: 16,890 entries). All MaxQuant parameters were set to default values. Trypsin was selected as the digestion enzyme; a maximum of two missed cleavages was allowed. For SILAC, peptide pairs were quantified by setting Lys6 as the heavy label. Enzyme specificity was set to LysC with an additional allowance of cleavage N-terminal to proline.

Methionine oxidation and N-terminal acetylation were set as variable modifications; carbamidomethylation of cysteines was chosen as a fixed modification. Except for the SILAC-labeled samples, the label-free quantification (LFQ) algorithm was used to quantify the measured peptides and the match between runs option was enabled to quantify peptides with a missing MS2 spectrum. A minimum ratio count of 2 was used for SILAC quantification.

Statistical analysis was performed using Perseus (1.5.5.3-1.6.5.0) software. Potential contaminants and reverse peptides were excluded, and values were log_2_ transformed. Two-sided *t*-tests were used to identify differentially expressed proteins between genotypes (*Perm1*^*−/−*^ or wild type) or conditions (immunoprecipitation or control). Identified peptides were annotated with the following Gene Ontology terms: Biological Process, Molecular Function, Cellular Compartment, and Kyoto Encyclopedia of Genes and Genomes terms. Graphical visualizations were achieved using Instant Clue software^54^.

### Western blot analysis

Protein lysates (10–20 µg) were incubated with 4x Laemmli buffer and 50 mM DTT for 10 min at 70 °C and SDS-PAGE was performed according to standard procedures using stain-free casting solutions from Bio-Rad. Proteins were electroblotted onto polyvinylidene fluoride (PVDF) membranes using a Trans-Blot Turbo Transfer System (Bio-Rad). Unblotted gels were developed using the ChemiDoc™ Touch Imaging System (Bio-Rad) with the stain-free option. Blotted membranes were developed using Clarity Western ECL Substrate (Bio-Rad). The intensities of the bands were quantified and normalized to the stain-free image using ImageJ software (version 1.8.0)^55^.

### Complexome profiling of mitochondria

Complexome profiling of mitochondria isolated from heart and muscle tissue was performed as previously described^56^. Freshly isolated mitochondria were lysed under non-denaturing conditions with 1% digitonin, 100 µg protein samples were loaded onto native gradient gels to separate the intact proteins complexes using a buffer containing Coomassie, as previously described^57^. Each gel lane was cut into 70 pieces and prepared for mass spectrometry analysis by in-gel digestion. Raw data were analyzed using iBAQ and individual profiles were analyzed with the NOVA tool (version 0.5.7)^28^.

### Oxygen consumption measurements

Oxygraphic measurements were performed on 40 µg of isolated muscle mitochondria as previously described^14^. The Oxygraph-2k (Oroboros Instruments) was used to measure oxygen consumption rate. The mitochondria were added after air equilibration of the mitochondrial respiration buffer. First, the OXPHOS state for complex I was determined by adding 5 mM pyruvate, 2 mM malate, 10 mM glutamate, and 2 mM ADP. Convergent electron flow through complexes I and II was measured by adding 10 mM succinate. Next, LEAK respiration was assessed by inhibition of the ATP synthase with oligomycin (1.5 µg/mL). Carbonyl cyanide-p- trifluoromethoxy-phenylhydrazone (FCCP, 0.5 µM) was titrated to determine maximal electron transfer system (ETS) capacity. Subsequently, complex I was inhibited by the addition of 0.5 µM rotenone to determine the maximal ETS capacity with electron flow through complex II only. Finally, residual oxygen consumption (ROX) was measured by inhibiting complex III using 2.5 µM antimycin A. Analysis was performed using the DatLab software (version 7.4.0.4; Oroboros Instruments.

### Determination of relative mtDNA copy number

DNA was extracted from tissue lysates and qPCR was performed as previously described^14^ to determine the relative mtDNA content of the soleus muscle tissues of *Perm1*^*−/−*^ mice. DNA was extracted from tissue lysates by incubation at 55 °C overnight in 350 µl DNA extraction buffer (0.5% SDS, 0.1 M NaCl, 50 mM Tris/HCl pH 8.0, 20 mM EDTA) supplemented with proteinase K. 350 µL phenol-chloroform-isoamylalcohol was added to the samples, mixed gently, and centrifuged for 5 min at 500 *g* and 4 °C. The upper phase was transferred to a new tube and 350 µL phenol-chloroform-isoamylalcohol was added, mixed gently, and centrifuged as before. The upper phase was transferred to a new tube, mixed with 650 µL of 100% ethanol and 65 µL of 7.5 mM ammonium acetate, incubated at −20 °C for 1 h. centrifuged at 15,000 *g* for 30 min at 4 °C, and the pellet was washed with 70% ethanol and resuspended in 10 mM Tris/HCl pH 8.5.

The extracted DNA (25 ng) was subsequently used for qPCR with two technical replicates. The expression of the cytochrome B gene (primers: GCTTTCCACTTCATCTTACCATTT and TGTTGGGTTGTTTGATCCTG) was normalized to the nuclear beta-actin gene (primers: GGAAAAGAGCCTCAGGGCAT and GAAGAGCTATGAGCTGCCTGA). The qPCR reactions were performed using KiCqStart^®^ SYBR^®^ Green qPCR ReadyMix™ (Sigma) on a CFX connect real-time PCR detection system (Bio-Rad) at 95 °C for 7 min, followed by 35 cycles of 95 °C for 10 s and 60 °C for 30 s. Data were collected using the CFX Manager™ Software for Bio-Rad CFX Real-Time PCR systems (version 3.0). Primers are listed in Supplementary Table 1.

### Determination of mitochondrial membrane potential in FDB fibers

Mitochondrial membrane potential was measured in isolated fibers FDB muscles as previously described^58^. FDB myofibers were placed in 1 mL Tyrode’s buffer and loaded with 2.5 nM TMRM supplemented with 1 µM cyclosporine H (a P-glycoprotein inhibitor) for 30 min at 37 °C. Sequential images of TMRM fluorescence were acquired every 60 s using a 20× magnification 0.5 numerical aperture UPLANSL objective (Olympus). Oligomycin (Olm, 5 µM) was added after 3 min and the protonophore carbonyl cyanide *p*-trifluoromethoxy-phenylhydrazone (FCCP, 4 µM) was added after 50 min. Images of TMRM fluorescence were acquired, stored, and analyzed over mitochondrial regions of interest using ImageJ software (1.8.0).

### Electron microscopy

Electron microscopy studies were performed with the assistance of the CECAD imaging facility. Tissues were dissected, fixed overnight in 2.5% glutaraldehyde, dehydrated in an ascending acetone series, embedded in epoxy resin, sectioned using an ultramicrotome, mounted on a grid and examined using a JEOL JEM2100PLUS electron microscope.

### Statistics and reproducibility

Statistical tests used for the individual experiments are provided in the figure legends of the respective figure. If not stated differently, immunoblottings, immunoprecipitations, immunohistochemical staining experiments, and in-vitro studies were conducted at least two times independently. All attempts of replication were successful.

### Reporting summary

Further information on research design is available in the Nature Research Reporting Summary linked to this article.

## Supplementary information

SI Figures

Supplementary Data 1

Supplementary Data 2

Supplementary Data 3

Supplementary Data 4

Supplementary Data 5

Description of additional supplementary files

Reporting Summary

## Data Availability

The mass spectrometry proteomics data have been deposited to the ProteomeXchange Consortium via the PRIDE partner repository^59^ with the dataset identifier PXD025745. All unique materials generated are readily available from the authors. Source data are provided with this paper.

## Source data

Source Data
