# Supplementary material for: PERM1 interacts with the MICOS-MIB complex to connect the mitochondria and sarcolemma via ankyrin B
Source: Nat Commun. 2021 Aug 12;12:4900. doi: 10.1038/s41467-021-25185-3 (PMC8361071; doi:10.1038/s41467-021-25185-3)
Supplement: Supplementary file 1 — SI Figures [file 41467_2021_25185_MOESM1_ESM.pdf]

Name	Purpose	Target vector	Forward primer	Reverse primer
Perm1_FLAGtag	plasmid generation for expression of Perm1 in cell culture	pcDNA5/TO_MCS_FLAGtag	AAAGGGAAAGGTACCATGGACAACTTCCAGTACAG	TTTCCCTTTCTCGAGGCAGCTGGGGTTTGA
Perm1_dTM	deletion of transmembrane domain	pcDNA5/TO_Perm1_FLAGtag	GCCCCCTCTCAGATCTACAAGCCCCAGATGC	GATCTGAGAGGGGGCTCCGTAGTTCC
mtDNA_b_actin	mtDNA copy number qPCR	/	GGAAAAGAGCCTCAGGGCAT	GAAGAGCTATGAGCTGCCTGA
mtDNA_cytb	mtDNA copy number qPCR	/	GCTTTCCACTTCATCTTACCATTT	TGTTGGGTTGTTTGATCCTG

Supplementary Table 1. List of primers.

Detailed list of all primers used in this study.

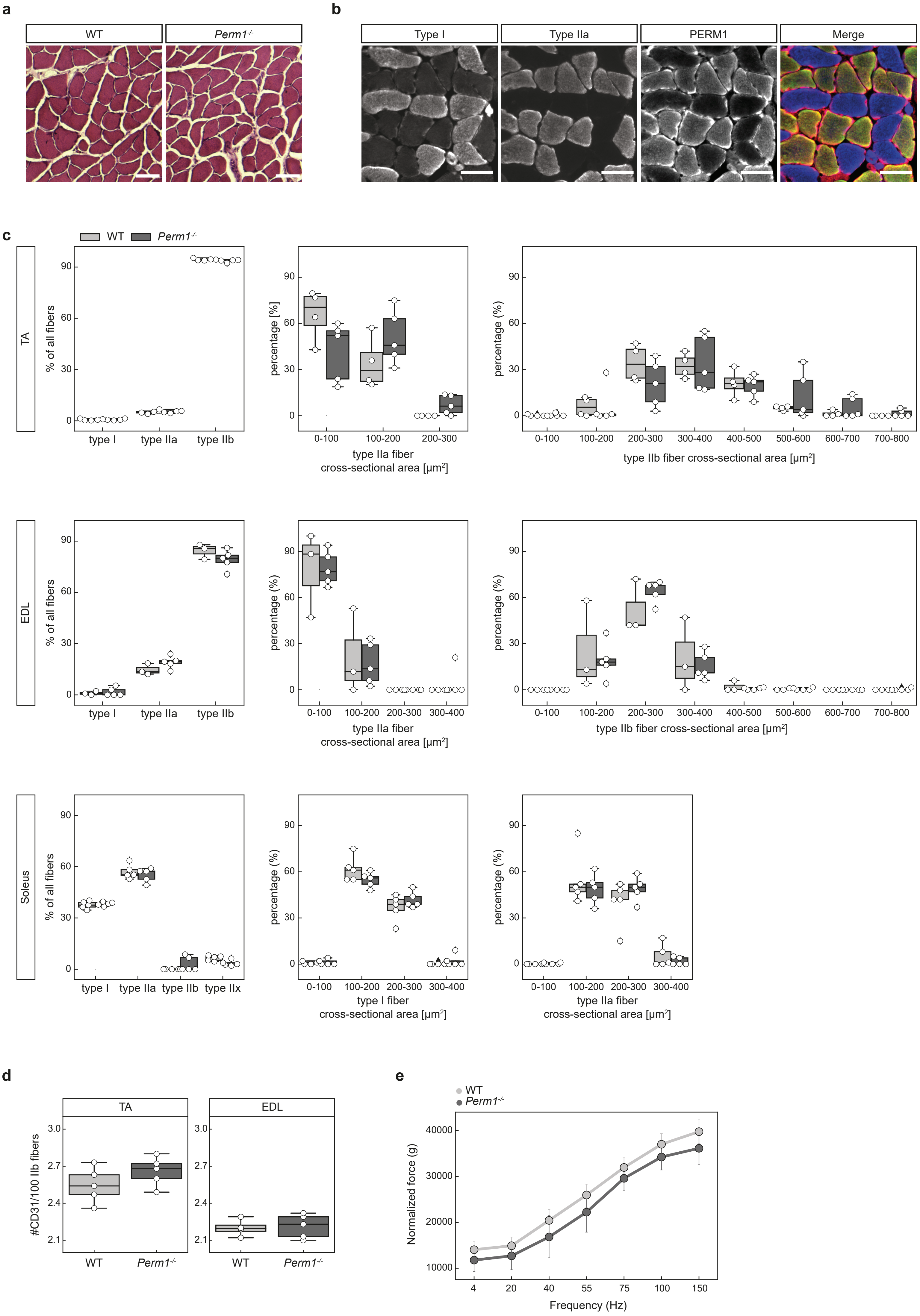

a
WT
Perm1-/-
b
Type I
Type IIa
PERM1
Merge
c
WT
Perm1-/-
TA
EDL
Soleus
% of all fibers
percentage [%]
percentage (%)
type I
type IIa
type IIb
type IIx
type IIa fiber cross-sectional area [μm²]
type IIb fiber cross-sectional area [μm²]
type I fiber cross-sectional area [μm²]
d
TA
EDL
#CD31/100 IIb fibers
WT
Perm1-/-
e
WT
Perm1-/-
Normalized force (g)
Frequency (Hz)

Supplementary figure 1. Deletion of *Perm1* causes no changes in fiber-type composition and cross-sectional area. (a) Hematoxylin and eosin (H&E) staining of mouse soleus cross sections demonstrated no changes between *Perm1*$^{-/-}$ mice and wild-type controls. Scale, 50 µm. (b) Immunohistochemical staining of soleus muscles of wild-type mice using myosin heavy chain-specific antibodies MyHC-I (type I fibers, blue) and MyHC-IIa (type IIa fibers, green) and anti-PERM1 (red) revealed localization of PERM1 predominantly in type IIa fibers. Scale, 50 µm. (c) Quantification of (left) fiber-type composition (right) and fiber cross-sectional area [μm^2] in the TA (top), EDL (middle) and soleus (bottom) muscles of *Perm1*$^{-/-}$ and wild-type mice based on staining with myosin heavy chain specific antibodies (*n*=3-5 mice per group). (d) Quantification of the number of capillaries per 100 type IIb fibers in the TA (left) and EDL (right) muscles of *Perm1*$^{-/-}$ and wild-type mice based on staining with a CD31/PECAM antibody (*n*=5 mice per group). (e) Force generated by the gastrocnemius muscle of *Perm1*$^{-/-}$ and wild-type control mice after electrical stimulation normalized for muscle mass at indicated frequencies (*n*=8 mutant mice; *n*=12 wild-type mice). *p*-value (Genotype) = 0.09, *p*-value (Frequency) = 1.6E-13, *p*-value (Frequency x Genotype) = 6.0E-04; two-way ANOVA.

Box plots (c+d) represent the median, 25th, and 75th percentiles, maximum and minimum are connected through whiskers, and individual data points are added on top. Outliers are defined as Q_1–1.8 IQR and Q_3+1.8 IQR. Data (e) are presented as mean values ± 0.95 CI. Source data are provided as Source Data file.

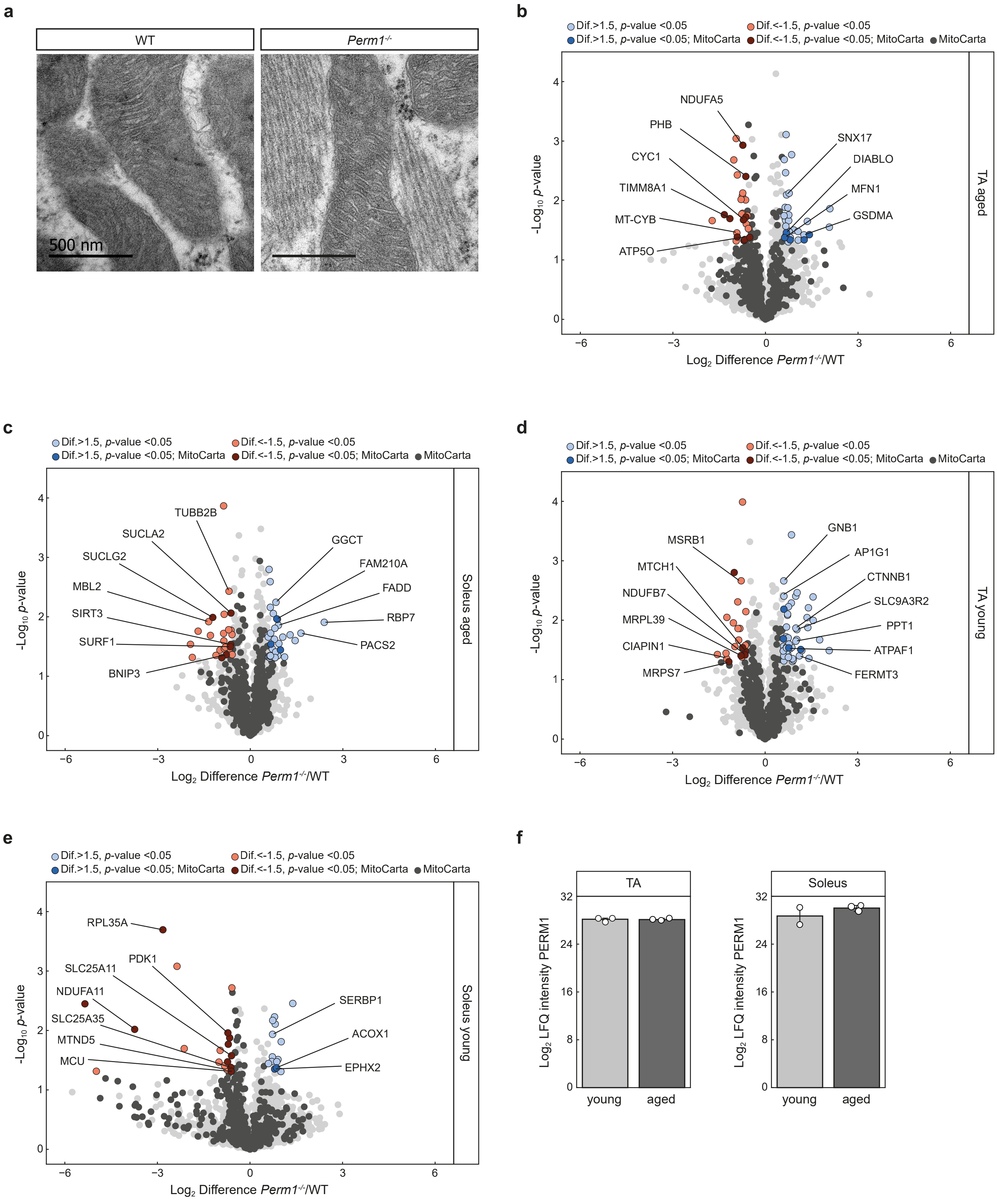
a
WT
Perm1-/-
500 nm
b
Dif.>1.5, p-value <0.05
Dif.<-1.5, p-value <0.05
Dif.>1.5, p-value <0.05; MitoCarta
Dif.<-1.5, p-value <0.05; MitoCarta
MitoCarta
NDUFA5
PHB
CYC1
TIMM8A1
MT-CYB
ATP5O
SNX17
DIABLO
MFN1
GSDMA
TA aged
-Log10 p-value
Log2 Difference Perm1-/-/WT
c
TUBB2B
SUCLA2
SUCLG2
MBL2
SIRT3
SURF1
BNIP3
GGCT
FAM210A
FADD
RBP7
PACS2
Soleus aged
d
MSRB1
MTCH1
NDUFB7
MRPL39
CIAPIN1
MRPS7
GNB1
AP1G1
CTNNB1
SLC9A3R2
PPT1
ATPAF1
FERMT3
TA young
e
RPL35A
PDK1
SLC25A11
NDUFA11
SLC25A35
MTND5
MCU
SERBP1
ACOX1
EPHX2
Soleus young
f
TA
Soleus
Log2 LFQ intensity PERM1
young
aged

Supplementary Figure 2. Cristae structure and proteomic analysis of young and aged whole TA and soleus muscle tissues from *Perm1*-deficient mice. (a) Electron microscopic analysis of TA muscle showing intact mitochondrial cristae structures in *Perm1*$^{-/-}$ mice and wild-type mice. Scale, 500 nm. (b-e) Volcano plots depicting significantly regulated proteins in *Perm1*$^{-/-}$ (b+c) TA and soleus muscle tissues (d+e) of (b+d) 3-month-old and (c+e) 24-month-old mice compared to wild-type mice (TA young n=3 mice per group; TA aged n=3 mice per group; soleus young n=2 mice per group; soleus aged n=3 mice per group). Cut-off was defined as $p < 0.05$ with a fold change >1.5. Darker colors indicate the MitoCarta 2.0 annotations. (f) Comparison of the $\log_2$ LFQ intensities of PERM1 obtained in young and aged TA and soleus muscles from *Perm1*$^{-/-}$ and wild-type mice. PERM1 LFQ intensity was based on 27 unique peptides (TA young n=3 mice per group; TA aged n=3 mice per group; soleus young n=2 mice per group; soleus aged n=3 mice per group).

(b-e) unpaired two-sided Student's *t*-test, S_0=0.1. Data (f) are presented as mean values ± 0.95 CI. Source data are provided as Source Data file and in Supplementary Data S2.

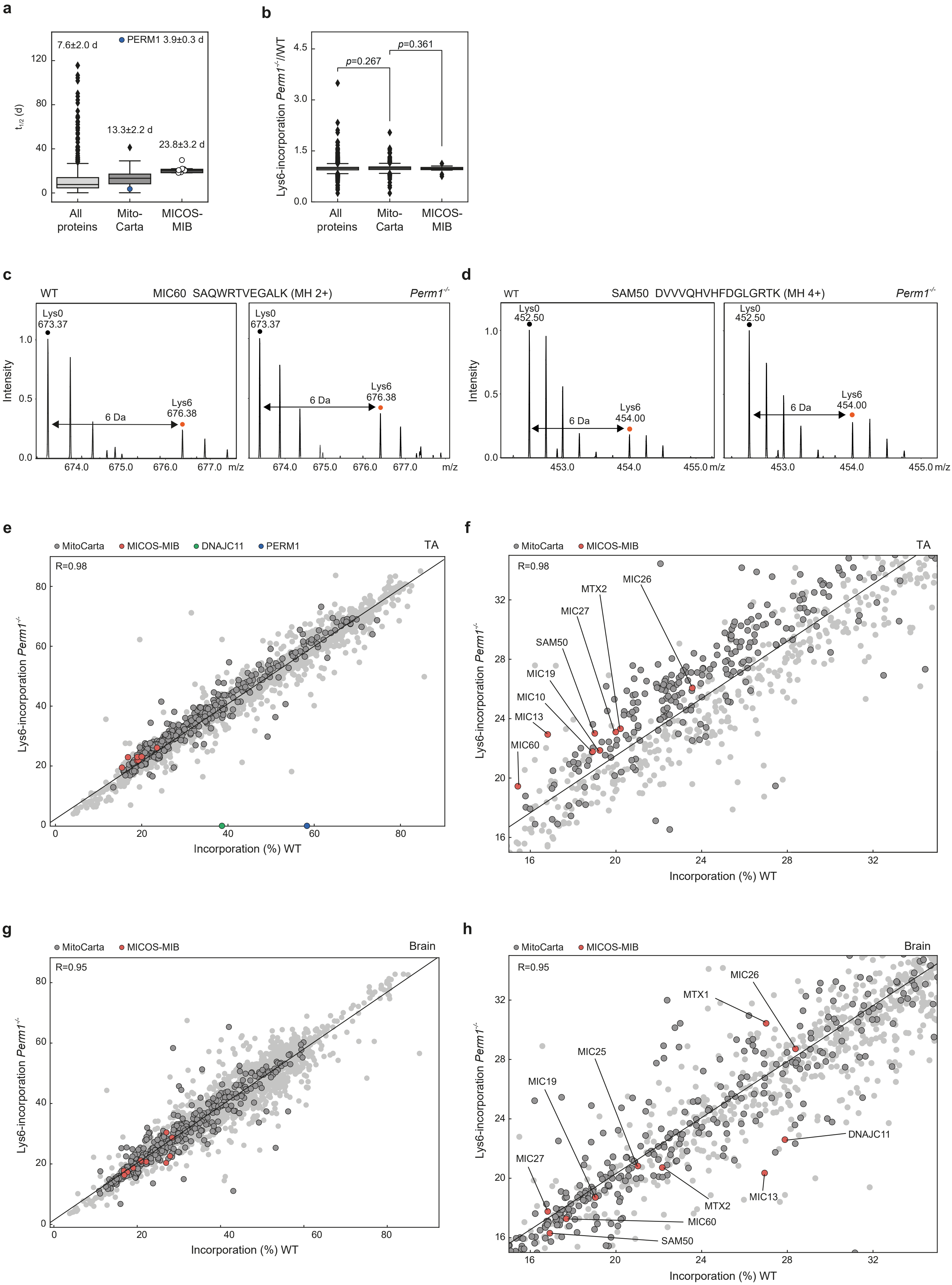
a
7.6±2.0 d
PERM1 3.9±0.3 d
13.3±2.2 d
23.8±3.2 d
t1/2 (d)
All proteins
Mito-Carta
MICOS-MIB
b
Lys6-incorporation Perm1-/-/WT
p=0.267
p=0.361
All proteins
Mito-Carta
MICOS-MIB
c
WT
MIC60 SAQWRTVEGALK (MH 2+)
Perm1-/-
Lys0 673.37
Lys6 676.38
6 Da
Intensity
m/z
d
WT
SAM50 DVVVQHVHFDGLGRTK (MH 4+)
Perm1-/-
Lys0 452.50
Lys6 454.00
6 Da
Intensity
m/z
e
MitoCarta
MICOS-MIB
DNAJC11
PERM1
TA
R=0.98
Lys6-incorporation Perm1-/-
Incorporation (%) WT
f
MitoCarta
MICOS-MIB
TA
R=0.98
MIC26
MTX2
MIC27
SAM50
MIC19
MIC10
MIC13
MIC60
Lys6-incorporation Perm1-/-
Incorporation (%) WT
g
MitoCarta
MICOS-MIB
Brain
R=0.95
Lys6-incorporation Perm1-/-
Incorporation (%) WT
h
MitoCarta
MICOS-MIB
Brain
R=0.95
MIC26
MTX1
MIC25
MIC19
DNAJC11
MIC27
MIC13
MTX2
MIC60
SAM50
Lys6-incorporation Perm1-/-
Incorporation (%) WT

Supplementary Figure 3. pSILAC-labelling of TA and brain tissues. (a) Calculated half-lives of mitochondrial and MICOS-MIB proteins in soleus muscles of mice fed with a Lys diet for 1-4 weeks (*n*=3 mice per timepoint). (b) Lys6-incorporation rates of MitoCarta-annotated proteins and MICOS-MIB complex-associated proteins were comparable in *Perm1*$^{-/-}$ mice and wild-type controls fed a SILAC diet for 14 days (*n*=3 mice per group). (c+d) MS- spectra of MIC60 (c) and SAM50 (d) in wild-type and *Perm1*$^{-/-}$ TA muscle depicting light (Lys0) and heavy (Lys6) labeled peptides from mice fed a SILAC diet for 14 days. (e-h) Scatter plots of the ratios of *Perm1*$^{-/-}$ to wild-type control Lys6-incorporation in (e+f) TA muscle and (g+h) brain tissue; proteins of the MICOS-MIB complex are highlighted. The Lys6-incorporation rates for PERM1 and DNAJC11 were retroactively added to the *x*-axis in (e), since these proteins were exclusively detected in wild-type muscles.

Box plots (a+b) represent the median, 25th, and 75th percentiles, maximum and minimum are connected through whiskers. Outliers are defined as Q_1–1.8 IQR and Q_3+1.8 IQR. (b) unpaired two-sided Mann-Whitney *U*-test. Source data are provided in Supplementary Data S3+S5.

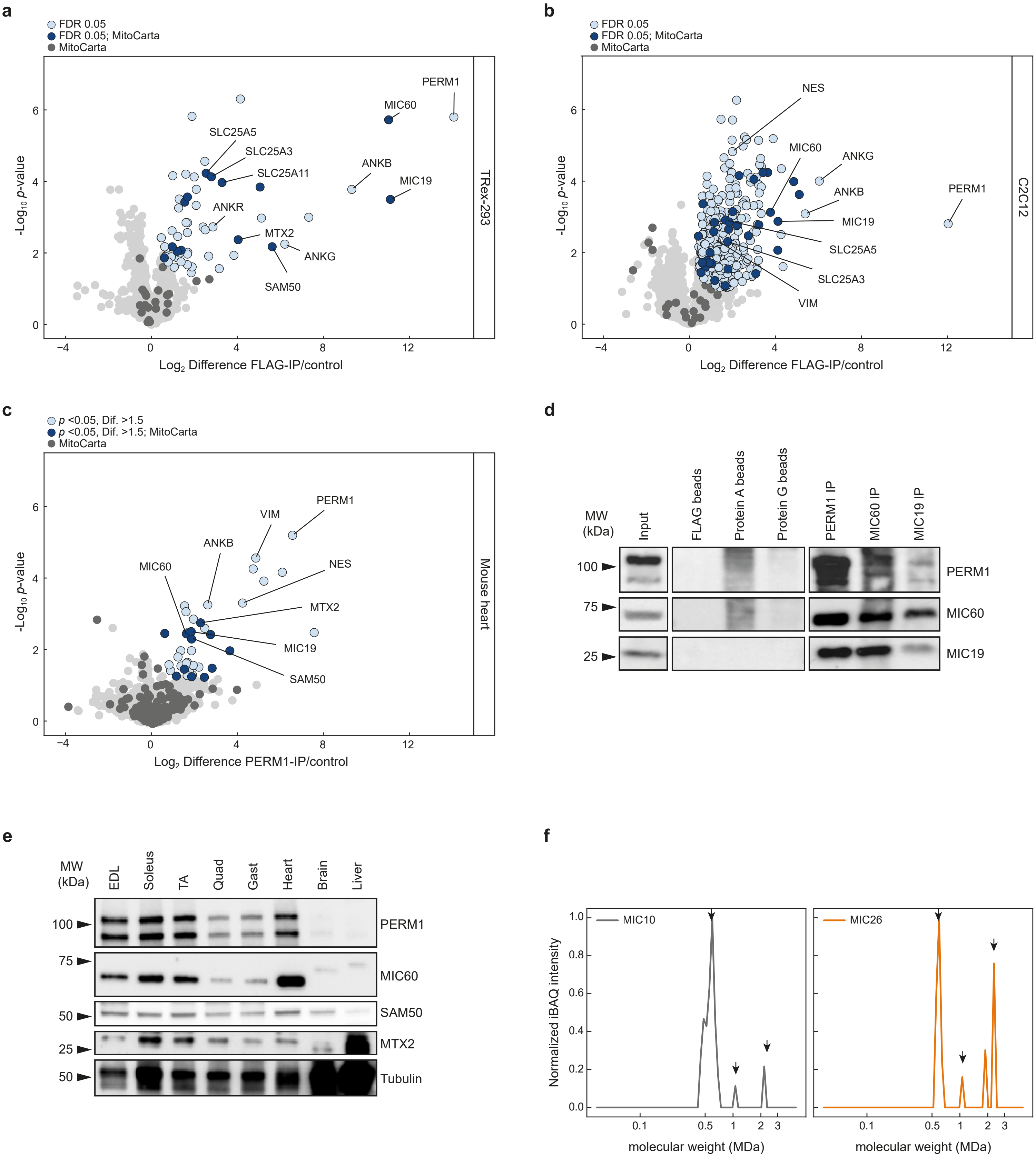
a
FDR 0.05
FDR 0.05; MitoCarta
MitoCarta
PERM1
MIC60
SLC25A5
SLC25A3
SLC25A11
ANKB
MIC19
ANKR
MTX2
ANKG
SAM50
TRex-293
-Log10 p-value
Log2 Difference FLAG-IP/control
b
FDR 0.05
FDR 0.05; MitoCarta
MitoCarta
NES
MIC60
ANKG
ANKB
PERM1
MIC19
SLC25A5
SLC25A3
VIM
C2C12
-Log10 p-value
Log2 Difference FLAG-IP/control
c
p <0.05, Dif. >1.5
p <0.05, Dif. >1.5; MitoCarta
MitoCarta
PERM1
VIM
ANKB
MIC60
NES
MTX2
MIC19
SAM50
Mouse heart
-Log10 p-value
Log2 Difference PERM1-IP/control
d
MW (kDa)
Input
FLAG beads
Protein A beads
Protein G beads
PERM1 IP
MIC60 IP
MIC19 IP
100
75
25
PERM1
MIC60
MIC19
e
MW (kDa)
EDL
Soleus
TA
Quad
Gast
Heart
Brain
Liver
100
75
50
25
50
PERM1
MIC60
SAM50
MTX2
Tubulin
f
MIC10
MIC26
Normalized iBAQ intensity
molecular weight (MDa)
molecular weight (MDa)

Supplementary Figure 4. Various PERM1 immunoprecipitation approaches and complexome profiling of heart mitochondria. (a-c) Volcano plots depicting significantly enriched proteins after immunoprecipitation of PERM1 in (a) TREx-293 cells stably expressing *Perm1*-FLAG, (b) undifferentiated C2C12 cells transfected with *Perm1*-FLAG, and (c) heart from wild-type mice. (*n*=3 samples per group and experiment). (d) Co-immunoprecipitation of *Perm1*-FLAG, MIC60, and MIC19 in TREx-293 cells stably overexpressing *Perm1*-FLAG and subsequent Western blotting using antibodies against PERM1, MIC60, and MIC19. (e) Protein levels of PERM1, MIC60, SAM50, and MTX2 in various mouse muscles and organs. (f) Complexome profiling of muscle mitochondria. Profiles of MIC10 and MIC26; iBAQ-values were maximum normalized. Arrows indicate the three main complex assemblies.

(a,b) unpaired two-sided Student's *t*-test, S_0=0.1, permutation-based FDR=0.05, 500 randomizations. (c) unpaired two-sided Student's *t*-test, S_0=0.1. Source data are provided as Source Data file and in Supplementary Data S4.
