# Supplementary material for: PERM1 interacts with the MICOS-MIB complex to connect the mitochondria and sarcolemma via ankyrin B
Source: Nat Commun. 2021 Aug 12;12:4900. doi: 10.1038/s41467-021-25185-3 (PMC8361071; doi:10.1038/s41467-021-25185-3)
Supplement: Supplementary file 7 — Description of additional supplementary files [file 41467_2021_25185_MOESM7_ESM.docx]

**Description of additional supplementary files**

**Title: Supplementary Data 1**

Description: **isolated IFM and SSM, related to Figure 2.** (IFM_SSM_TA_heart) Protein groups output table of lysates from isolated IFM and SSM from TA and heart tissues of *Perm1^-/-^* and wild-type mice (*n*=3 mice per group). Unpaired two-sided Student’s *t*-test, S_0_=0.1.

**Title: Supplementary Data 2**

**Description: TA, soleus and isolated crude mitochondrial proteomes, related to Figure 3b+d-e and SI Figure S2b-f.** (proteome_whole_TA_11pm) Protein groups output table of lysates from TA muscle tissues of *Perm1^-/-^* and wild-type mice isolated at 11 pm, related to Figures 3b+d-e (*n*=2 mice per group). Unpaired two-sided Student’s *t*-test, S_0_=0.1. (proteome_crude_mitochondria_11pm) Protein groups output table of lysates from crude mitochondria isolated from TA muscle tissues of *Perm1^-/-^* and wild-type mice isolated at 11 pm, related to Figure 3d (*n*=2 mice per group). Unpaired two-sided Student’s *t*-test, S_0_=0.1.(proteomes_young_aged_TA_soleus) Protein groups output table of lysates from TA and soleus muscle tissue of 3-month-old (young) and 24-month-old (aged) *Perm1^-/-^* and wild-type mice, related to SI Figure S2b-f (TA young *n*=3 mice per group; TA aged *n*=3 mice per group; soleus young *n*=2 mice per group; soleus aged *n*=3 mice per group). Unpaired two-sided Student’s *t*-test, S_0_=0.1.

**Title: Supplementary Data 3**

**Description: Lys6-incorporation rates, related to Figure 4e-h and SI Figure S3.** Protein groups output table of lysates from TA and brain tissues of *Perm1^-/-^* and wild-type mice fed a SILAC diet for 14 days (*n*=3 mice per group).

**Title: Supplementary Data 4**

Description: **the various PERM1 immunoprecipitations, related to Figure 5a+b+h and SI Figure S4a-c.** (FLAG-IP TRex-293) Immunoprecipitation of PERM1 in TREx-293 cells stably overexpressing FLAG-tagged *Perm1*, related to SI Figure S4a. TREx-293 cells stably overexpressing an empty vector were used as control (*n*=3 samples per group). Unpaired two-sided Student’s *t*-test, S_0_=0.1, permutation-based FDR=0.05, 500 randomizations (FLAG-IP C2C12) Immunoprecipitation of PERM1 in C2C12 myoblasts transiently transfected with FLAG-tagged *Perm1*, related to SI Figure S4b. C2C12 myoblasts transfected with an empty vector were used as control (*n*=3 samples per group). Unpaired two-sided Student’s *t*-test, S_0_=0.1, permutation-based FDR=0.05, 500 randomizations (PERM1-IP_Mouse_TA) Immunoprecipitation of endogenous PERM1 in isolated TA muscles of wild-type mice, related to Figure 5a. As a control, beads were incubated without addition of the PERM1 antibody (*n*=3 samples per group). Unpaired two-sided Student’s *t*-test, S_0_=0.1. (PERM1-IP_Mouse_heart) Immunoprecipitation of endogenous PERM1 in isolated heart tissues of wild-type mice, related to SI Figure S4c. As a control, beads were incubated without addition of the PERM1 antibody (*n*=3 samples per group). Unpaired two-sided Student’s *t*-test, S_0_=0.1. (IP transmembrane mutant) Immunoprecipitation of PERM1 in HEK-293T cells transfected with FLAG-tagged wild-type *Perm1* and FLAG-tagged ΔTM mutant, related to Fig. 5h (*n*=3 samples per group). Unpaired two-sided Student’s *t*-test, S_0_=0.1, permutation-based FDR=0.05, 500 randomizations

**Title: Supplementary Data 5 for Lys6 incorporation rates of soleus muscles related to Figure S3a.**

Description: **Lys6 incorporation rates of soleus muscles related to Figure S3a.** (Lys6_incorporation_soleus) Protein groups output table of lysates from soleus muscles of mice fed a SILAC diet for 7, 14, 21, or 28 days (*n*=3 mice per time point). (half-lives) Calculation of t_1/2_ (d) of lysates from soleus muscles of mice fed a SILAC diet for 7, 14, 21, or 28 days (*n*=3 mice per time point) according to ^60^.

**Title: Supplementary Data 6.**

Description: **List of primers.** Detailed list of all primers used in this study.
