# Supplementary material for: PERM1 interacts with the MICOS-MIB complex to connect the mitochondria and sarcolemma via ankyrin B
Source: Nat Commun. 2021 Aug 12;12:4900. doi: 10.1038/s41467-021-25185-3 (PMC8361071; doi:10.1038/s41467-021-25185-3)
Supplement: Supplementary file 8 — Reporting Summary [file 41467_2021_25185_MOESM8_ESM.pdf]

nature research

Corresponding author(s): Marcus Krüger

Last updated by author(s): Jun 28, 2021

Reporting Summary

Nature Research wishes to improve the reproducibility of the work that we publish. This form provides structure for consistency and transparency in reporting. For further information on Nature Research policies, see Authors & Referees and the Editorial Policy Checklist.

Statistics

For all statistical analyses, confirm that the following items are present in the figure legend, table legend, main text, or Methods section.

n/a	Confirmed	
☐	☒	The exact sample size (*n*) for each experimental group/condition, given as a discrete number and unit of measurement
☐	☒	A statement on whether measurements were taken from distinct samples or whether the same sample was measured repeatedly
☐	☒	The statistical test(s) used AND whether they are one- or two-sided<br>*Only common tests should be described solely by name; describe more complex techniques in the Methods section.*
☒	☐	A description of all covariates tested
☐	☒	A description of any assumptions or corrections, such as tests of normality and adjustment for multiple comparisons
☐	☒	A full description of the statistical parameters including central tendency (e.g. means) or other basic estimates (e.g. regression coefficient) AND variation (e.g. standard deviation) or associated estimates of uncertainty (e.g. confidence intervals)
☐	☒	For null hypothesis testing, the test statistic (e.g. *F*, *t*, *r*) with confidence intervals, effect sizes, degrees of freedom and *P* value noted<br>*Give P values as exact values whenever suitable.*
☒	☐	For Bayesian analysis, information on the choice of priors and Markov chain Monte Carlo settings
☒	☐	For hierarchical and complex designs, identification of the appropriate level for tests and full reporting of outcomes
☐	☒	Estimates of effect sizes (e.g. Cohen's *d*, Pearson's *r*), indicating how they were calculated

Our web collection on statistics for biologists contains articles on many of the points above.

Software and code

Policy information about availability of computer code

Data collection	Xcalibur (version 3.1); DatLab 7.4.0.4 (Oroboros Instruments); CFX Manager™ Software for Bio-Rad CFX Real-Time PCR Systems (version 3.0); Muscle lever system (Model 305C; Aurora Scientific, Aurora, ON, Canada)
Data analysis	MaxQuant analysis software and the implemented Andromeda software (1.5.3.8); Perseus (1.5.5.3-1.6.5.0); Instant Clue (0.4.9-0.5.2); ImageJ (1.8.0); CellProfiler (3.1.9), NOVA (0.5.7)

For manuscripts utilizing custom algorithms or software that are central to the research but not yet described in published literature, software must be made available to editors/reviewers. We strongly encourage code deposition in a community repository (e.g. GitHub). See the Nature Research guidelines for submitting code & software for further information.

Data

Policy information about availability of data

All manuscripts must include a data availability statement. This statement should provide the following information, where applicable:

- Accession codes, unique identifiers, or web links for publicly available datasets
- A list of figures that have associated raw data
- A description of any restrictions on data availability

The mass spectrometry proteomics data have been deposited to the ProteomeXchange Consortium via the PRIDE partner repository with the dataset identifier PXD025745 (https://www.ebi.ac.uk/pride/archive/projects/PXD025745). All unique materials generated are readily available from the authors. Source data are provided with this paper.

Field-specific reporting

Please select the one below that is the best fit for your research. If you are not sure, read the appropriate sections before making your selection.

☒ Life sciences ☐ Behavioural & social sciences ☐ Ecological, evolutionary & environmental sciences

For a reference copy of the document with all sections, see nature.com/documents/nr-reporting-summary-flat.pdf

Life sciences study design

All studies must disclose on these points even when the disclosure is negative.

Sample size	Based on our previous experiments with the Perm1 mouse models we used biological duplicates/triplicates for immunoblotting and the proteomics analysis (Aravamudhan et al., 2021; JMCC). For the running experiments, weight tests and force measurements, we used at least 8-12 animals per group (Müller et al. 2014; Front. Physiol.). Based on previous experience from similar protein-protein interaction studies, all IPs were performed in biological triplicates to confirm reproducibility (Vukotic et al., 2017; Mol Cell). QPCR analysis was performed in biological triplicates and the electron microscopic inspection of TA muscles was conducted in biological duplicates. Similar, IFM and SSM fractions were analyzed and measured in biological triplicates. In vivo labeling with stable isotopes in mice was performed in biological triplicates. Oroboros O2 flux measurements were performed in biological triplicates.<br>No statistical calculations were used to determine sample size a priori. Sample sizes are indicated in each figure legend.
Data exclusions	During analysis of muscle cross-sections using the CellProfiler, incorrectly recognized fibers within the image mask were manually excluded from the data set. Other than that, no data was excluded from the analyses.
Replication	For the in vivo studies, we analyzed a sufficient number of animals (at least 2 mice per group) to ensure that the results show a consistent difference between controls and mutants. All other experiments (stainings, IPs, etc.) were replicated at least two times individually, as reported in the method section. All attempts of replication were succesful.
Randomization	No specific method of randomization had been used to select animals since age and sex were relevant in our studies. We did not specifically control for covariates in this study. All samples for the mass spectrometric analysis were performed in randomized order to avoid batch effects during the measurement time. For in vitro experiments, randomization was irrelevant to the design of the experiments and was therefore not performed.
Blinding	Histological analysis of muscle tissue sections was performed blindly. Except for histological analysis no blinding to group allocation was done during data collection and/or analysis as this was not practical for experiments performed by a single investigator.

Reporting for specific materials, systems and methods

We require information from authors about some types of materials, experimental systems and methods used in many studies. Here, indicate whether each material, system or method listed is relevant to your study. If you are not sure if a list item applies to your research, read the appropriate section before selecting a response.

Materials & experimental systems

n/a	Involved in the study	
☐	☒	Antibodies
☐	☒	Eukaryotic cell lines
☒	☐	Palaeontology
☐	☒	Animals and other organisms
☒	☐	Human research participants
☒	☐	Clinical data

Methods

n/a	Involved in the study	
☒	☐	ChIP-seq
☒	☐	Flow cytometry
☒	☐	MRI-based neuroimaging

Antibodies

Antibodies used	The antibodies used for immunoblotting, immunoprecipitation and immunohistochemistry were: PERM1 (Sigma, #HPA031711), diluted 1:1,000 for immunoblotting, 1:100 for immunohistochemistry; BNIP3 (Cell Signaling, #3769), diluted 1:1,000; TOM20 (Abcam, #ab56783), diluted 1:100 for immunohistochemistry; TOM20 (Sigma, #HPA011562), diluted 1:5,000 for immunoblotting, 1:100 for immunohistochemistry; GAPDH (Invitrogen, #AM4300), diluted 1:10,000 for immunoblotting; Ankyrin B (Invitrogen, #33-3700), diluted 1:1000 for immunoblotting, 1:100 for immunohistochemistry; MYH7 (Developmental Studies Hybridoma Bank [DSHB], BA-F8), MYH2 (DSHB, SC-71), MYH4 (DSHB, BF-F3), CD31/PECAM (BD Pharmingen, #553370), all diluted 1:100 for immunohistochemistry; anti-FLAG M2-HRP (Sigma, #A8592), anti-HA-HRP (Miltenyi, #130-091-972), Alexa Fluor 350 anti-mouse IgG2b (Life Technologies, #A-21140), Alexa Fluor 488 anti-mouse IgG1 (Life Technologies, #A-21121), Alexa Fluor 546 anti-mouse IgM (Life Technologies, #A-21045), Alexa Fluor 546 anti-rabbit (H+L) (Life Technologies, #A-11010), Alexa Fluor 488 anti-rat (Life Technologies, #A-11006), anti-mouse HRP (Sigma, #A9044) and anti-rabbit HRP (Sigma, #A0545), all diluted 1:4,000 for immunoblotting and 1:200 for immunohistochemistry.

Validation	In immunohistochemistry experiments, negative controls were used to account for non-specific staining by using secondary antibody only. Biologically negative samples were used whenever possible.<br>All commercially available antibodies have been validated by the vendor for the target and application, with publications and validation information indicated on the manufacturer's websites for the respective antibody. Vendors used are: Sigma, CST, Abcam, Invitrogen, DSHB, BD Pharmingen, Miltenyi and Life Technologies with the calalog numbers listed in the methods section.<br><br>PERM1: https://www.sigmaaldrich.com/DE/de/product/sigma/hpa031711?context=product<br>BNIP3: https://www.cellsignal.com/products/primary-antibodies/bnip3-antibody-rodent-specific/3769<br>TOM20 4F3: https://www.abcam.com/tomm20-antibody-4f3-bsa-and-azide-free-ab56783.html<br>TOM20: https://www.sigmaaldrich.com/DE/de/product/sigma/hpa011562?context=product<br>GAPDH 6C5: https://www.thermofisher.com/antibody/product/GAPDH-Antibody-clone-6C5-Monoclonal/AM4300<br>Ankyrin B 2.2: https://www.thermofisher.com/antibody/product/Ankyrin-B-Antibody-clone-2-2-Monoclonal/33-3700<br>MYH7/BA-F8: https://dshb.biology.uiowa.edu/BA-F8<br>MYH2/SC-71: https://dshb.biology.uiowa.edu/SC-71<br>MYH4/BF-F3: https://dshb.biology.uiowa.edu/BF-F3<br>CD31/PECAM MEC 13.3: https://www.citeab.com/antibodies/2408223-553370-bd-pharmingen-purified-rat-anti-mouse-cd31<br>anti-FLAG M2-HRP: https://www.sigmaaldrich.com/DE/de/product/sigma/a8592?context=product<br>anti-HA-HRP GG8-1F3-3-1: https://www.miltenyibiotec.com/DE-en/products/ha-antibody-gg8-1f3-3-1.html?countryRedirected=1#hrp:100-ul<br>Alexa Fluor 350 anti-mouse IgG2b: https://www.thermofisher.com/antibody/product/Goat-anti-Mouse-IgG2b-Cross-Adsorbed-Secondary-Antibody-Polyclonal/A-21140<br>Alexa Fluor 488 anti-mouse IgG1: https://www.thermofisher.com/antibody/product/Goat-anti-Mouse-IgG1-Cross-Adsorbed-Secondary-Antibody-Polyclonal/A-21121<br>Alexa Fluor 546 anti-mouse IgM: https://www.thermofisher.com/antibody/product/Goat-anti-Mouse-IgM-Heavy-chain-Cross-Adsorbed-Secondary-Antibody-Polyclonal/A-21045<br>Alexa Fluor 546 anti-rabbit (H+L): https://www.thermofisher.com/antibody/product/Goat-anti-Rabbit-IgG-H-L-Cross-Adsorbed-Secondary-Antibody-Polyclonal/A-11010<br>Alexa Fluor 488 anti-rat: https://www.thermofisher.com/antibody/product/Goat-anti-Rat-IgG-H-L-Cross-Adsorbed-Secondary-Antibody-Polyclonal/A-11006<br>anti-mouse HRP: https://www.sigmaaldrich.com/DE/de/product/sigma/a9044?context=product<br>anti-rabbit HRP: https://www.sigmaaldrich.com/DE/de/product/sigma/a0545?context=product

Eukaryotic cell lines

Policy information about cell lines

Cell line source(s)	C2C12, undifferentiated mouse myoblasts (ATCC)<br>T-REx-293 TO5 Flp-In T-REx-293 stably expressing TO5-STOP-FLAG (in-house development)<br>T-REx-293 PERM1 Flp-In T-REx-293 stably expressing PERM1-FLAG (in-house development)<br>HEK-293T cells (ATCC)
Authentication	PERM1 expression in T-Rex-293-Perm1 was confirmed by immunoblotting. All other cell lines appeared as expected when subjected to light microscopy, no other authentication method was used.
Mycoplasma contamination	All cell lines were tested negative for mycoplasma contamination.
Commonly misidentified lines<br>(See ICLAC register)	No commonly misidentified cell lines were used

Animals and other organisms

Policy information about studies involving animals; ARRIVE guidelines recommended for reporting animal research

Laboratory animals	Perm1 mice were generated in cooperation with the Max Plank Inst. for Heart and Lung Research. Constitutive knockout Perm1-/- mice were generated using a recombineering based method to subclone DNA from bacterial artificial chromosomes (BACs) into high-copy plasmids, together with Flp recombinase to introduce FRT sites into the subcloned DNA. In the first step, two sets of primers were used to amplify two ~500 bp regions of the BAC (obtained from mouse bMQ BAC library, Source BioScience). The region of the BAC containing Venus-GFP, Neo cassette and the FRT sites was excised and transformed into ES cells (129sv). The Neo positive clones and recombination positive clones of ES cells were confirmed using southern blotting and one was chosen and inject into C57BL6 mouse. Chimeric mice were crossed with Flp recombinase mice to cut out the Neo cassette between the FRT sites. Further breeding and back crossings are being performed to generate heterozygous and homozygous mice and remove the Flp deletor gene. The resulting mice have a mixed background of C57BL6/NRj C57BL/6 JRi C57BL/6J.<br>Mice that were used for proteomics experiments were age and sex-matched for proteomics, only age-matched for all other experiments. If not explicitly stated differently, mice were between 3-9 months old. Mice were housed at 22 ± 2 °C, with a humidity of 55 ± 10%, and an air exchange rate of 15 times per hour on a 12 h light-dark cycles with free access to food and water.
Wild animals	The study did not involve wild animals
Field-collected samples	The study did not involve samples collected from the field.

Ethics oversight | All mouse experiments were performed in accordance with institutional guidelines and according to regulations of the LANUV.

Note that full information on the approval of the study protocol must also be provided in the manuscript.
